# Overview of Systematic Reviews on Factors Related to the Structure and Functioning of Residential Long-Term Care Facilities for Older Adults

**DOI:** 10.3390/geriatrics10030064

**Published:** 2025-05-03

**Authors:** Aurélio Matos Andrade, Karine Rodrigues Afonseca, Tatiana de Almeida Jube, Suelen Meira Góes, Maíra Catharina Ramos, Flavia Tavares da Silva Elias

**Affiliations:** 1 Program of Evidence for Health Policy and Technologies (PEPTS), Fiocruz Brasília, Oswaldo Cruz Foundation (Fiocruz), Asa Norte, Brasilia 70904-130, DF, Brazil; maira.ramos@fiocruz.br (M.C.R.); flavia.elias@fiocruz.br (F.T.d.S.E.); 2 Health Surveillance Department, State Health Department of the Federal, District SEPS-Qd. 712/912, Bl. D, Asa Sul, Brasilia 70390-125, DF, Brazil; karine.afonseca@gmail.com; 3 Agência Nacional de Vigilância Sanitária (Anvisa), SIA-Trecho 5-Área Especial 57-Bloco D, Brasilia 71205-050, DF, Brazil; tatiana.jube@anvisa.gov.br; 4 Saskatchewan Health Quality Council, Innovation Place, The Atrium, 111 Research Dr, Saskatoon, SK S7N 2X8, Canada; su.goes@gmail.com

**Keywords:** aged, homes for the aged, terminal care, health of institutionalized elderly

## Abstract

**Objective:** To identify factors influencing the structure and functioning of long-term residential care facilities for older adults worldwide, in order to uncover practices and support evidence-based improvements in care delivery. **Method:** An overview of systematic reviews was performed according to the PRISMA protocol and registered on the PROSPERO platform (no. CRD42023486204). Research was carried out on 21 September 2023, using the following databases: PubMed (via MedLine), EMBASE, Web of Science, Scopus, Virtual Health Library (VHL), and Epistemonikos. **Results:** The search yielded 12,040 articles, including 61 systematic reviews. Analyzing the primary outcomes, personnel structure, and risk management were the most-studied outcomes of the systematic reviews, followed by pharmaceuticals, food services, mobility/accessibility, and technological and physical structures. In terms of primary outcomes of the systematic reviews, the personnel structure was the most highlighted (in 39.34%), followed by risk management (in 32.79%), while the least highlighted was physical structure (in 9.84%). **Conclusions:** Personnel are critical to the safe and effective functioning of Long-Term Care Facility (LTCF) operations. Future research is needed to identify associations between models of care and structural concerns, including physical environment, as they relate to quality of care in LTCFs, particularly in low and middle-income countries (LMIC).

## 1. Introduction

Population aging is a growing reality in most countries around the world. According to the United Nations (UN), in 2021, there were 761 million people aged 65 or over. The projection is that, by 2050, this number will reach 1.6 billion [1].

It is known that older adults with complex needs may demand care that many families do not have the human, financial, or social support resources to provide. Long-Term Care Facilities (LTCFs) have emerged to address the need for comprehensive care for older adults who cannot access this individualized or collective support at home, or for families/older adults who opt for group living arrangements [2,3].

It is important to understand that families encounter difficulties arising from their own social transformation, such as new family arrangements, separations between spouses, the migration of younger people, and the participation of women in the workforce. These factors contribute to the search for support in LTCFs, day centers, assisted living technologies, or residential areas designed for older adults. Such facilities meet the LTC needs in multiple aspects, preserving the human dignity of older adults [3,4].

Long-term care services are structured in different ways around the world, but with very similar objectives: meeting the basic needs of care and social coexistence of older adults who, with advancing age and the acquisition of chronic diseases, experience significant limitations in performing basic activities independently, such as bathing, dressing, and eating. It is understood that an individual setting for the older adult in LTCF compromises human dignity; with this in mind, more and more countries are faced with the need to improve and expand LTCF services to better serve older adults in a pluralistic way [5,6].

The structure and functioning of LTCFs are components that ensure the quality of care offered to residents and are influenced by organizational factors. The structure refers to the physical, human, and normative configuration, the availability of material resources, and the adequacy of the environment to the needs of the elderly, including accessibility, safety, and comfort [7]. The functioning involves care processes and practices, institutional management, workflows, and the implementation of person-centered care protocols [8]. These elements are interrelated to the extent that an adequate structure favors the operationalization of more effective and humanized practices. According to the quality assessment model proposed by Donabedian [7], the structure and process directly influence health outcomes, which is particularly relevant in long-term care contexts, such as LTCFs. Furthermore, more integrated organizational models, with a focus on interdisciplinarity and the active participation of the elderly and their families, contribute to better indicators of well-being, functionality, and satisfaction with care [9].

In this sense, it is understood that LTCFs can be public, private, or philanthropic institutions, serving as collective residential facilities for older adults with or without family support, ultimately providing conditions of freedom, dignity, and citizenship [5]. In the USA and Australia, the majority of LTCFs are privately run and receive resources or tax benefits from the government; meanwhile, in Brazil, most LTCFs are philanthropic. Regardless of the form of management/financing model, it has been observed worldwide that LTCF services for older adults are expensive, with increasing demand and sometimes insufficient physical structures and work processes to meet the demands of older adults and their families [6,10].

Living in an LTCF is an alternative for a part of the world’s older adult population who are unable to live independently; it may also represent the only means of access to healthcare, social support, and security, ensuring quality of life for older adults [2]. It is worth noting that the 2030 Agenda and the Sustainable Development Goals (SDGs) recognize that development will only be possible if it is inclusive for those of all ages [11].

Given the complexity of services and the needs of older adults with physical and cognitive needs and priorities, several countries have improved their frameworks for regulating these services. Conversely, numerous studies are being conducted to define the minimum requirements necessary for older adults to live in environments that support care and human dignity [2]. The aim of this overview of systematic reviews was to identify factors influencing the structure and functioning of long-term residential care facilities for older adults worldwide, in order to uncover practices and support evidence-based improvements in care delivery.

## 2. Method

### 2.1. Study Identification

This study comprises an overview of systematic reviews with and without meta-analysis, structured according to the PRISMA guidelines and registered on the PROSPERO platform under the code CRD42023486204 (Supplementary File Table S1). The research question was structured according to the PICOS acronym, described as follows: “What are the structural and functional factors of care for older adults residing in long-term residential care facilities?” (Table 1).

The search was carried out using the PubMed (via MedLine), EMBASE, Web of Science, Scopus, Virtual Health Library (VHL), Cochrane Library and Epistemonikos databases on 21 September 2023, using the Boolean operators “AND” and “OR” with the following Health Sciences Descriptors (DeCS) and Medical Subject Headings (MeSH): “Aged”, “Health of Institutionalized Elderly”, “Homes for the Aged”, “Health Services for the Aged”, “Terminal Care”, “Risk Management”, “Wound Infection”, “Pressure Ulcer”, “Patient Safety”, “Food Handling”, “Diet Therapy”, “Social Capital”, “Social Infrastructure”, “Architectural Accessibility”, “Mobility Limitation”, “Workforce”, “Pharmaceutical Services” and “Hygiene”. Research in the gray literature was not considered relevant, due to the comprehensive scientific and academic scope of the studies available in these databases (Supplementary File Table S2).

In this overview, reference lists of included studies were manually reviewed, and records of Randomized Clinical Trials (RCTs) from reviews with meta-analyses were checked. In addition, experts in the field of gerontology were consulted by email.

### 2.2. Eligibility Criteria

The inclusion criteria were systematic review studies with or without meta-analysis, with no restrictions on time or language, that addressed structural and functional factors of care for older adults living in long-term residential or community care facilities.

The exclusion criteria were studies that referred to older adults in association with children, adolescents and adults; studies that focused on care for older adults in individual residences or homes without connections to public or private institutions; and studies that addressed chronic diseases such as diabetes mellitus, hypertension, cancer, depression or other mental disorders in a specific way without discussing the requirements or models of residential aged care facilities (Supplementary File Table S3).

This research adopted the definition of the World Health Organization (WHO), which considers the age threshold for older adult individuals based on the socioeconomic level of each nation: starting at 65 years old in high-income countries, and 60 years old in low- or middle-income countries [12]. The definition of long-term stay was considered to refer to a period that could last for the rest of an individual’s life [13].

### 2.3. Study Selection and Data Collection

The Mendeley 1.18^®^ reference manager was used to organize articles and remove duplicates, and the Rayyan QCRI^®^ virtual platform was used to select articles (by title and abstract) identified in searches of scientific databases by two independent researchers (A.M.A. and K.R.A.). Disagreements were resolved by a third researcher (M.C.R.), acting independently.

### 2.4. Data Extraction

Data extraction was carried out independently by the authors (A.M.A. and K.R.A.) using a Microsoft Excel 2016^®^ spreadsheet. Discrepancies were resolved by consensus. Data extraction included the following variables: authors, year of publication, country of publication, country where the establishment is located, type of establishment, objective, age group, management financing model, study funding, and description of structural and functional factors.

As primary outcomes, factors related to the follow structures were determined: (1) personnel structure (team composition by professional categories), (2) physical structure, (3) technological structure; and functional factors: (4) risk management (infection, falls and pressure injuries), (5) diet/food services, (6) pharmaceutical assistance; and (7) mobility/accessibility, adopted globally for older adults residing in LTCFs. Management financing models and the types of services provided (social assistance and/or health) by LTCFs were defined as secondary outcomes.

### 2.5. Quality Assessment

Quality assessment of the included studies was performed using the AMSTAR 2 tool through the website https://amstar.ca/Amstar_Checklist.php (accessed on 1 December 2023). The AMSTAR 2 tool evaluates the methodological quality of systematic reviews (with or without meta-analysis) that include randomized and non-randomized studies [14]. The tool comprises the following critical domains: Protocol registration prior to start of the review; adequacy of bibliographical research; justification for excluded studies; risk of bias for included studies; adequacy of meta-analytic methods; consideration of the risk of bias when interpreting results; and assessment of the presence and potential impact of publication bias. From this assessment, it is possible to classify the methodological quality of systematic reviews (with or without meta-analysis) as high, moderate, low, or critically low.

## 3. Results

**Figure 1:**
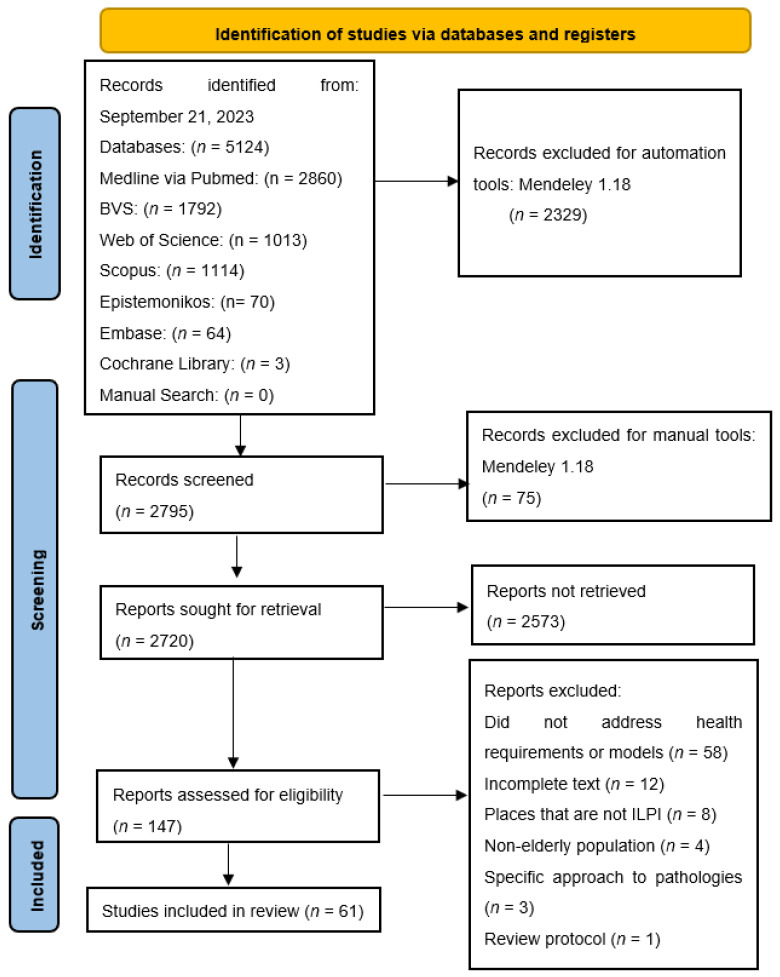
Article selection flowchart adapted based on the PRISMA statement (2020).

A total of 147 articles were selected for full-text reading. After complete reading, 86 were excluded as they did not meet the eligibility criteria, leaving 61 systematic reviews (Figure 1).

The publications occurred over the last 17 years, with emphasis on 2019 as the year with the highest number of publications (9) (14.75%). The countries with the most publications were Australia (19.67%) and the United Kingdom (19.67%). In contrast, no publications were identified in Latin America or the African continent (Table 2). The primary studies of the systematic reviews highlighted the United States of America (USA) (7.64%) as the leading study location, followed by the United Kingdom (3.96%) and Canada (3.39%) for long-term care residential facilities for older adults. Latin America was not a study location in any of the primary studies (Supplementary File Table S4).

The most-used terminology in 33 systematic reviews to determine the type of establishment was “Long-Term Care Facilities (LTCF)” [18,19,24,25,27,28,29,30,31,32,33,35,37,39,41,45,49,50,51,52,58,59,62,63,64,67,69,70,72,73,74,75,76,77], followed by “Nursing Homes” (25 reviews) [15,16,20,21,22,23,26,34,38,40,42,43,46,47,48,50,56,57,60,65,69,71,73] and “Residential Aged Care (RAC)” (9 reviews) [17,27,36,44,50,52,61,62,66] (Table 2). Age groups of 65 years or older were reported in 27.87% of studies [17,19,23,26,27,31,33,37,44,49,53,57,58,60,61,69], while 60 years or older was noted in 26.23% [30,36,40,41,47,50,51,73]; however, the majority of studies (45.90%) did not specify the age group [15,16,18,20,21,22,24,25,28,29,32,34,35,37,38,39,42,43,45,47,48,52,54,55,56,59,62,63,64,65,66,67,68,70,71,72,74,75].

In the secondary outcomes, the management model was verified in the primary studies: private law for-profit establishments (14.45%), private non-profit establishments (6.80%), and public law establishments (7.37%). However, most studies (71.38%) did not report the management model. The type of service provided was focused on health in all systematic reviews, while social assistance was explicitly mentioned in only one systematic review (Supplementary File Table S4).

The quality assessment, carried out using AMSTAR 2, classified low (17) and critically low-quality reviews (39) as comprising 91.80% of the sample, while high- (1) and moderate-quality reviews (4) totaled 8.20% (Supplementary File Table S5). Approximately 1/6 were systematic reviews with meta-analysis, and almost half (47.54%) of systematic reviews did not obtain funding for research development (Table 2).

**Figure 2:**
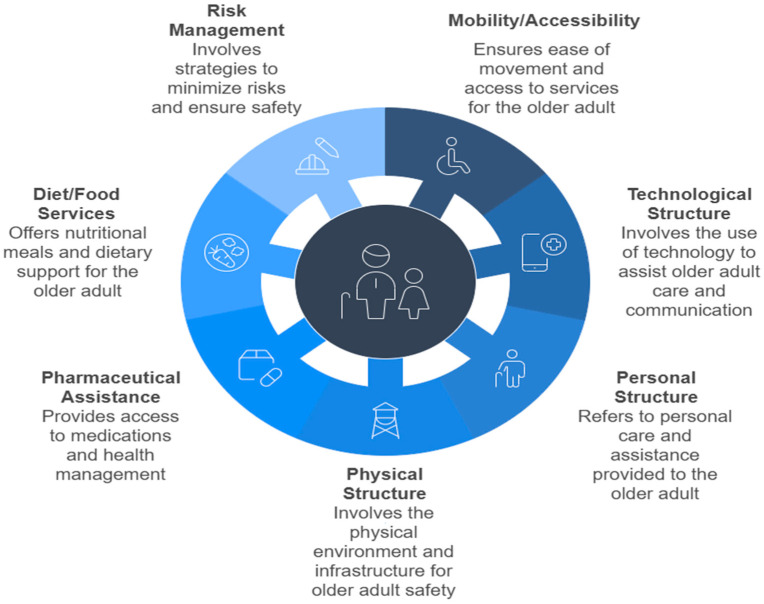
Factors related to the structure and functioning adopted globally for older adults in LTCFs.

In analyzing the primary outcomes: Mobility/accessibility was evidenced in 13.11% [22,43,46,56,68,69,73,75]; technological structure in 13.11% [25,32,35,43,48,50,73,75]; personnel structure in 39.34% [15,17,18,20,24,31,33,34,38,45,47,48,49,50,51,54,57,61,62,64,67,72,73,74]; physical structure in 9.84% [29,33,48,60,71,73]; pharmaceutical assistance in 18.03% [21,27,40,41,42,52,54,62]; diet/food services in 16.39% [18,19,23,31,32,41,49,50,53,70]; and risk management in 32.79% [16,18,26,28,30,34,36,37,39,43,44,49,55,58,59,63,65,66,71] of systematic reviews (Figure 2).

Mobility/accessibility was associated with exercise/physical activity and exergames aimed at strengthening muscles, balance, flexibility, autonomy, and functional capacity [22,43,46,56,68,69,73,75]. The technological structure included telemedicine using audio and video technologies, digital gaming platforms, mobile radiography services, mobile devices (tablets and smartphones), Resident Assessment Instrument (RAI), interactive art, and virtual cycling [25,32,35,43,48,50,73,75]. The personnel structure emphasized the contributions of various professional categories, including nursing (62.50%), pharmacy (25%), medicine (12.5%), physiotherapy (12.5%), social assistance (8.33%), dentistry (8.33%), nutrition (4.17%), occupational therapy (4.17%) and psychology (4.17%). More than one professional category was identified in a single review [15,17,18,20,24,31,33,34,38,45,47,48,49,50,51,54,57,61,62,64,67,72,73,74]. Pharmaceutical assistance included medication registration, administration, communication, prescription, review, reduction, suspension, and management [21,27,40,41,42,52,54,62]. The diet/food services indicated criteria regarding high protein and fluid intake, calcium and vitamin D supplementation, real food diet, nutritional assessments, and specific diets for subgroups of older adults [18,19,23,31,32,41,49,50,53,70]. Risk management included physical exercises, hip protectors, bed rails to reduce falls, Personal Protective Equipment (PPE), vaccination programs, algorithms, and programs to prevent pressure injuries, urinary tract infections, or falls. Additional measures included pillows and mattresses to reduce pressure injuries, mass testing, and increasing the number of available beds to mitigate infections and deaths due to COVID-19. Strategies also targeted respiratory infections and multidrug-resistant organisms (MDROs), including biosafety measures to reduce methicillin-resistant Staphylococcus aureus (MRSA) contamination [16,18,26,28,30,34,36,37,39,43,44,49,55,58,59,63,65,66,71] (Supplementary File Table S6).

## 4. Discussion

The findings indicate a growing interest in publications related to long-term care institutions for older adults, particularly in 2019. This trend may reflect an increasing global recognition of aging populations and their associated healthcare needs (United Nations, 2019) [76]. However, the lack of studies in Latin America and Africa highlights a significant gap in the literature. This omission is concerning, given the rapid aging rates in regions such as sub-Saharan Africa and the implications for developing tailored care models [77].

The terminology used to define care institutions varied significantly, with “Long-Term Care Facilities” being the most common. While this diversity may reflect regional preferences or differences in translation, it underscores the need for standardized definitions in research to facilitate global comparisons. Uniformity in terminology could improve data synthesis and policy alignment across nations [78].

Mobility and accessibility interventions, such as exercise programs and assistive devices, were strongly associated with improved functional outcomes. This finding corroborated existing evidence that physical activity enhances mobility, reduces falls, and improves overall quality of life among older adults [79]. Expanding access to these interventions, especially in resource-limited settings, could yield significant health benefits. Baumann et al. [80] highlighted that residents of LTCFs experience significant improvements in mobility through regular physical activity. This benefit is amplified when skilled professionals are available, as well as when family and friends provide support and encouragement. Many LTCFs, particularly those in low-resource settings, lack the necessary infrastructure or trained personnel to implement effective exercise interventions [81].

The use of digital games, such as exergames, has proven to be an effective strategy for enhancing cognitive and motor functions in older adults living in LTCFs. These interactive tools foster engagement and satisfaction, making physical activity more appealing [82]. Additionally, interactive art and video resources have been beneficial in managing behavioral symptoms in residents with dementia; for example, these technologies can help to calm agitated residents and promote participation in structured activities [83]. Telehealth applications have also brought notable advantages to LTCFs, enabling access to specialist consultations in fields such as psychiatry and dermatology. This approach not only enhances clinical outcomes but also reduces costs associated with off-site consultations [84].

Corroborating Pitkälä et al. [85] and Emiri [86], skilled nursing care significantly reduces hospital transfers and improves outcomes in LTCFs, with pharmacists playing important roles in optimizing medication regimens. Geriatric dentistry is an emerging field gaining societal relevance in LTCFs, particularly considering the growing need for oral health monitoring in aging populations. Di Spirito et al. [87] highlighted the role of teledentistry in addressing these needs, enabling real-time surveillance and improved access to dental care. However, resource constraints, staff shortages, and limited training hinder the delivery of comprehensive care. Investment in training programs and multidisciplinary teams—including physical therapists, dietitians, and occupational therapists—can improve the quality of LTCF services.

Pharmaceutical care assumes a role in older adults’ health, particularly in addressing age-related pharmacokinetic and pharmacodynamic changes. Additionally, it contributes to maintaining pharmacotherapy, ensuring the safe and effective use of medications. Treatment adherence may be compromised by cognitive and sensory impairments, such as memory loss and visual decline, highlighting the pharmacist’s vital role. However, pharmaceutical follow-up alone is insufficient, and interdisciplinary strategies are essential to achieve better therapeutic outcomes [88].

The physical environment of LTCFs profoundly impacts the safety and mental health of residents. Features such as ramps, alarm systems, and natural elements (gardens, green spaces) reduce accidents and improve mental health outcomes, including lower levels of depression and anxiety [84]. However, implementing these physical environments often requires significant funding, which poses challenges for under-resourced facilities. Policymakers should prioritize infrastructural investments to ensure that all residents benefit from safe and therapeutic environments [89].

Malnutrition and sarcopenia are highly prevalent among older adults in LTCFs, with rates potentially reaching twice those observed in community-dwelling older adult populations. These conditions significantly contribute to muscle mass loss, increased frailty, and reduced life expectancy [90]. Addressing these challenges requires targeted interventions aimed at enhancing dietary intake and nutritional status in LTCFs; for instance, implementing regular nutritional assessments and individualized dietary plans can help to identify and mitigate the risks associated with malnutrition and sarcopenia, particularly in vulnerable populations. These findings highlight the importance of prioritizing structured nutritional support as an integral component of care for LTCF residents [32].

Despite the positive impacts of nutritional interventions, barriers such as limited resources, staff shortages, and a lack of training can impede the consistent delivery of high-quality dietary care in LTCFs. Addressing these challenges requires a systemic approach that includes increased investment in training programs, the adoption of evidence-based protocols, and the use of innovative solutions, such as food fortification or meal delivery services tailored to the specific needs of residents [89]. Strengthening the infrastructure for dietary and nutritional services can significantly enhance the overall quality of care and health outcomes for older adults in LTCFs.

The detailed exploration of risk management strategies—including fall prevention and infection control—reflects a comprehensive approach to the care of older adults. The emphasis on multimodal strategies aligns with best practices for mitigating risks in long-term care settings [91]. However, the limited focus on psychosocial interventions indicates a need for restructuring in these care settings. Addressing mental health and social connectedness needs is equally vital to holistic care approaches for older adults [92].

### 4.1. Limitations

There are several limitations that warrant attention to advancing geriatric research in this overview. First, the over-representation of studies from high-income countries such as Australia, the United Kingdom, and the United States reflects an imbalance in research contributions, with low- and middle-income countries (LMICs) being significantly under-represented despite housing a substantial proportion of the older adult population. This disparity limits the global applicability of findings and calls for greater inclusion of LMIC settings to inform equitable policy development. Second, the under-reporting of specific age groups (as observed in 45.9% of studies) constitutes a methodological challenge, particularly when addressing the unique needs of the “older” population (≥85 years), who often require tailored interventions. Third, the predominance of systematic reviews of low or critically low quality, as assessed via AMSTAR 2, underscores the need for greater methodological rigor and adherence to reporting standards. Strengthening the quality of systematic reviews is necessary to generate reliable evidence that can directly inform best practices in geriatric care. Fourth, the variability of different cultural perspectives of LTCFs and funding sources influences the availability of resources for older adults. Finally, the failure to specify governance structures (e.g., private, public, non-profit) in most studies limits the ability to evaluate their influence on the quality of care, despite evidence suggesting that non-profit facilities often deliver superior outcomes.

### 4.2. Practical Implications

Practical implications include the need to improve the quality and efficiency of structural and functional factors characteristic of LTCFs for older adults. Understanding the recommendations that have impacted older adults during the COVID-19 pandemic is a first step toward making such changes, such as incorporating compartmentalization, allowing sick residents to be moved to the same compartment (as a “quarantine zone”); implementing syndromic surveillance in facilities with high population densities; providing care via telemedicine; and that hand sanitizing stations should not be limited to the entrance of LCTFs, but further distributed at different points throughout the LTCFs. Similarly, identifying factors associated with staffing levels, training, and care protocols can help to address challenges such as staffing shortages and inconsistencies in care delivery. Policymakers and facility managers can develop targeted strategies that improve resident well-being, ensure safety, and promote the sustainability of LTCFs.

## 5. Conclusions

This overview of systematic reviews highlighted critical factors that influence the design and operation of long-term care facilities (LTCFs) for older adults. Evidence supports the benefits of mobility interventions, digital technologies, and telehealth applications in improving residents’ functional outcomes, engagement, and access to specialized care. However, persistent barriers such as resource constraints, staff shortages, and limited training hinder the delivery of comprehensive care. Furthermore, the findings suggest that most studies related to standards and quality of care in LTCFs have focused on the effectiveness of interventions, with few being individual-centric, and were mainly conducted in European countries and the United States. Addressing issues such as malnutrition, sarcopenia, and the lack of psychosocial interventions requires a systemic approach that addresses multidisciplinary teams and facility infrastructure.

The evidence provided in this overview has the potential to guide policymakers and stakeholders to prioritize strategies that improve the quality of care and well-being of LTCF residents globally. Future studies focusing on the individual factors and the safety of older adults, particularly with respect to under-represented geographic settings such as low- and middle-income countries, are needed to inform standards and quality of care for those living in LTCFs.

## Figures and Tables

**Table 1:** PICOS definition of the systematic review.

PICOS	
P	Older adult population residing in institutions or specific long-term care spaces
I	Long-Term Care Facilities, among other synonyms
C	Not applicable
O	Classification of types of institutions and the need to structure some health services. Operational outcomes for critical points and attention: risk management (infection, falls, and pressure injuries) and dietary/food services, cleaning, and pharmaceutical assistance (excluding mental health). Structural outcomes: human resources, physics, technology, mobility/accessibility.
S	Systematic reviews with and without meta-analysis

Source: Authors’ elaboration.

**Table 2:** Characterization of systematic reviews (*n* = 61).

Author/ Year	Country of Publication	Objective	Type of Establishment	Quality of the Study *	Study Funding
Bostick et al., 2006 [15]	USA	Evaluate a range of staffing measures and data sources for long-term use in public reporting on staffing as a measure of quality in nursing homes.	Nursing homes	Critically Low-quality review	No financing
Sawka et al., 2006 [16]	Canada	Design a Bayesian random-effects model to pool binary outcome data from cluster randomized trials (CRTs) with individually randomized trials (IRTs), and then use this model to determine whether hip protectors decrease the risk of hip fracture in elderly people living in nursing homes.	Nursing homes	Critically Low-quality review	No financing
Dwyer et al., 2011 [17]	Australia	Critically evaluate, synthesize, and present the best available evidence about nurses’ experiences as clinical leaders and managers in residential aged care institutions.	Residential Aged Care	Critically Low-quality review	No financing
Neyens et al., 2011 [18]	Netherlands	Report the effectiveness and implementation aspects of interventions aimed at reducing falls in elderly residents of long-term care institutions: a systematic review of randomized controlled trials (RCTs).	Long-Term Care Facilities	Critically Low-quality review	No financing
Sinclair et al., 2011 [19]	UK	Summarize the evidence base from published studies in the area, and analyze documents and other materials relevant to long-term diabetes care in residential and nursing homes.	Long-Term Care Facilities	Critically Low-quality review	No financing
Bradshaw et al., 2012 [20]	UK	Conduct a systematic qualitative review of life in nursing homes and provide practical recommendations to improve the quality of life for residents.	Nursing homes	Critically Low-quality review	MS Society of Great Britain and Northern Ireland
Tamura et al., 2012 [21]	USA	To assess factors associated with polypharmacy and high risk of medication-related problems among community-dwelling older people in the Netherlands, Greece, Croatia, Spain, and the United Kingdom.	Nursing homes	Critically Low-quality review	The John A. Hartford Foundation Center of Excellence in Geriatrics, University of Hawaii
Valenzuela et al., 2012 [22]	Australia	Provide a synthesis of evidence from clinical trials to determine whether progressive resistance training, as a single exercise intervention, improves strength and functional performance in institutionalized older adults.	Nursing homes	Critically Low-quality review	No financing
Abbott et al., 2013 [23]	UK	Determine the effectiveness of mealtime interventions for older adults living in residential care and, where possible, determine which types of mealtime interventions were most effective.	Nursing homes	Low-quality review	National Institute forHealth Research through Peninsula CLAHRC
Donald et al., 2013 [24]	Canada	Report quantitative evidence of the effectiveness of advanced practice nursing roles, clinical nurse specialists, and nurse practitioners in meeting the health care needs of older adults living in long-term care residential settings.	Long-Term Care Facilities	Critically Low-quality review	National Institute for Health Research (NIHR) Oxford Biomedical Research Center (BRC)
Edirippulige et al., 2013 [25]	Australia	Systematically review the literature on the use of telemedicine in LTCFs and assess the quality of published evidence.	Long-Term Care Facilities	Critically Low-quality review	University of Queensland
Hughes et al., 2013 [26]	UK	Determine the effects of infection control strategies on preventing MRSA transmission in nursing homes.	Nursing homes	Critically Low-quality review	Research and Development Office, Northern Ireland, UK.NIHR/Department of Health (England), (Cochrane Wounds Group), UK
Reyes-Alcázar et al., 2013 [27]	Spain	Seek recommendations on patient safety based on scientific evidence with the aim, in different socio-sanitary contexts, of achieving safer care.	Long-Term Care Facilities; Nursing homes, Residential Aged Care; Accommodation for the elderly	Critically Low-quality review	Andalusian Society for Quality in Health
Silva et al., 2013 [28]	Australia	Analyze the impact and characteristics of the most effective physical exercise regimen to prevent falls and fractures in this specific scenario.	Long-Term Care Facilities	Critically Low-quality review	Nepean Medical Research Foundation and Science Without Borders CNPq Brazil Program
Van Malderen et al., 2013 [29]	Belgium	Systematically review the literature, focusing on identifying interventions that attempt to improve the QoL of residents of LTCF facilities.	Long-Term Care Facilities	Critically Low-quality review	No financing
Chan et al., 2014 [30]	China	Conduct a systematic review and meta-analysis for the effectiveness of influenza vaccination in institutionalized elderly people with measures to minimize these confounding factors.	Long-Term Care Facilities	Critically Low-quality review	No financing
Flanagan et al., 2014 [31]	UK	Review interventional studies that included UI and/or fecal incontinence (FI) which investigated factors associated with management (economic, skin care, exercise and mobility, staff quality, and continence promotion), but where management of incontinence techniques was not the main focus or outcome.	Long-Term Care Facilities	Critically Low-quality review	Wirral University Teaching Hospitals NHS Foundation Trust Research and Development Fund
Bunn et al., 2015 [32]	UK	To evaluate the effectiveness of interventions and environmental factors in increasing fluid intake or reducing the risk of dehydration in elderly people living in long-term care facilities.	Long-Term Care Facilities	Low-quality review	National Institute for Health Research (NIHR)
Liu et al., 2015 [33]	USA	To evaluate the effectiveness of interventions on the dietary performance of elderly people with dementia in long-term care facilities (LTCFs).	Long-Term Care Facilities	Critically Low-quality review	No financing
Low et al., 2015 [34]	Australia	Systematically identify and describe studies that investigated the effects of interventions to change staff practices or care approaches in order to improve outcomes for nursing home residents; identify interventions or intervention components that lead to successful team practices or changes in care approach in nursing homes; identify potential barriers and facilitators to team practice or change in care approach in nursing homes.	Nursing homes	Low-quality review	University of New South Wales, Queensland University of Technology, and Australian National University Collaborative Dementia Research Centres
Marasinghe et al., 2015 [35]	Canada	To evaluate the impact of Computerized clinical decision support systems (CCDSS) on improving medication safety in Long-Term Care Facilities (LTCFs).	Long-Term Care Facilities	Critically Low-quality review	No financing
Pagan et al., 2015 [36]	New Zealand	Establish the composition and effect of wound-related programs, implementation strategies, resident and clinical staff outcomes, and program sustainability in RAC facilities.	Residential Aged Care (RAC)	Critically Low-quality review	No financing
Vlaeyen et al., 2015 [37]	Belgium	To determine the characteristics and effectiveness of single, multiple, and multifactorial fall prevention programs on the number of falls and recurrent falls in older adults permanently residing in a nursing home.	Long-Term Care Facilities	Critically Low-quality review	Flemish Ministry of Welfare, Public Health and Family of Belgium and the University Derde Leeftijd Leuven vzw.
Alldred et al., 2016 [38]	UK	Determine the effect of interventions to optimize overall prescribing for older people living in nursing homes.	Nursing homes	Moderate quality review	School of Healthcare, University of Leeds, UK
Diehl et al., 2016 [39]	Norway	Systematically review the effects of interventions to improve the implementation of guidance in nursing homes.	Long-Term Care Facilities	Moderate quality review	No financing
Morin et al., 2016 [40]	Sweden	To systematically evaluate the prevalence of potentially inappropriate medication use in nursing home residents.	Nursing homes	Low-quality review	No financing
Thiruchelvam et al., 2016 [41]	Malaysia	Assess the impact of medication reviews in aged care facilities, with additional focus on types of medication reviews, using randomized controlled trials (RCTs) and observational studies.	Long-Term Care Facilities	Low-quality review	No financing
Ferrah et al., 2017 [42]	Australia	Determine the prevalence and characteristics of medication errors resulting in hospitalization and death of nursing home residents, and the factors associated with the risk of death and hospitalization.	Nursing homes	Critically Low-quality review	No financing
Kjelle et al., 2017 [43]	Norway	Identify the outcomes of mobile radiography services for nursing home residents and society at large.	Nursing homes	Critically Low-quality review	University College of Southeast Norway
Lee et al., 2017 [44]	South Korea	To evaluate the effectiveness of exercise interventions on the rate of falls and the number of falls in healthcare facilities.	Residential Aged Care	Low-quality review	Gachon University
Barker et al., 2018 [45]	UK	Systematically identify and synthesize evidence on which professional group should provide first-line medical care (routine and/or unscheduled) to LTCF residents to improve health outcomes.	Long-Term Care Facilities	Low-quality review	National Institute for Health Research, School of Primary Care Research, and NIHR Health Services and Delivery Research
Cao et al., 2018 [46]	China	Determine the effectiveness of exercise in preventing falls in nursing home residents.	Nursing homes	Critically Low-quality review	No financing
Clarkson et al., 2018 [47]	UK	Provide an overview of the range of services that have been provided and studied to address the following research questions: What are the main characteristics of variation in organization, activities, and responsibilities?	Nursing homes	Critically Low-quality review	National Institute for Health Research (NIHR); School of Social Care Research (SSCR)
Ghavarskhar et al., 2018 [48]	Iran	Determine the structure of modified and purpose-built housing, independent housing, residential care, and nursing homes in Iran and developed countries.	Nursing homes	Critically Low-quality review	Tabriz University of Medical Science
MäkiÿTurjaÿRostedt et al., 2018 [49]	Finland	Explore the effectiveness of preventive interventions for pressure ulcers (PUs) in long-term care facilities (LTCFs).	Long-Term Care Facilities	Low-quality review	Government research funds: Satakunta Hospital District and Turku University Hospital
Panza et al., 2018 [50]	Italy	To systematically review the body of evidence from the last three decades of clinical research dedicated to the implementation of broad geriatric assessment programs in LTCFs (i.e., nursing homes, residential homes, and rehabilitation facilities), analyzing the benefits arising from the application in these settings of tools based on CGA.	Long-Term Care Facilities: Nursing homes, Residential Aged Care	Critically Low-quality review	No financing
Brett et al., 2019 [51]	Australia	(1) When and how are physiotherapy services used by elderly people living in nursing homes? (2) What are the factors associated with the use of physical therapy services in nursing homes? (3) How are physical therapy services in nursing homes documented and monitored?	Long-Term Care Facilities	Low-quality review	No financing
Chen et al., 2019 [52]	Australia	To systematically review the literature reporting processes, impact, and outcomes of medication review and reconciliation in Australian residential aged care facilities (RACFs).	Long-Term Care Facilities	Critically Low-quality review	Cognitive Decline Partnership Center (CDPC). Australian Government. NHMRC Early Career. NHMRC Dementia Leadership Fellowship
Donaldson et al., 2019 [53]	UK	To evaluate the effectiveness of meat-free high-protein supplementation on health-related quality of life (HRQoL) and relevant clinical and nutritional outcomes in older adults in a home setting.	Nursing homes	Low-quality review	National Institute for Health Research (NIHR) Oxford Biomedical Research Center (BRC)
Huey Lee et al., 2019 [54]	Malaysia	Provide an overview of the evidence for pharmacist-led interventions to improve the quality of medication use in nursing homes and determine the impact of these interventions in nursing homes.	Long-Term Care Facilities	Critically Low-quality review	No financing
Hye Lee et al., 2019 [55]	South Korea	Review and analyze the effectiveness and components of infection prevention and control programs in LTCFs for the elderly.	Long-Term Care Facilities	Low-quality review	National Research Foundation of Korea (NRF) is funded by the government of Korea (Ministry of Science and ICT)
Maurer et al., 2019 [56]	Switzerland	Identify the attitudes and needs of nursing home residents regarding physical activity.	Nursing homes	Critically Low-quality review	No financing
Nguyen et al., 2019 [57]	UK	Evaluate the effect of interventions to improve antimicrobial stewardship in nursing homes and report the results used in these trials.	Nursing homes	Low-quality review	Vietnam International Education Cooperation Development in Vietnam
Schoberer et al., 2019 [58]	Austria	Provide a comprehensive collection of evidence on the effectiveness of exercise interventions to prevent falls and support clinical decision making.	Long-Term Care Facilities	Low-quality review	No financing
Senderovich et al., 2019 [59]	Canada	Summarize the current literature available on the effectiveness of the HZ vaccine in adults over 60 years of age living in LTCFs and evaluate the cost-effectiveness of the HZ vaccine.	Long-Term Care Facilities	Critically Low-quality review	No financing
van den Berg et al., 2020 [60]	Australia	Summarize reported barriers and facilitators to nursing home residents’ use of outdoor spaces.	Nursing homes	Critically Low-quality review	National Health and Medical Research Council (NHMRC) Partnership Centre
Wu et al., 2020 [61]	Australia	Explore the effectiveness of nurse-led interventions to prevent urinary tract infections in older adults living in residential aged care facilities.	Residential Aged Care	High-quality review	GriffithUniversity
Ali et al., 2021 [62]	Australia	To investigate the efficacy and effectiveness of pharmacist-led interventions to reduce adverse drug events (ADEs) in older people living in residential aged care facilities (RACFs).	Long-Term Care Facilities	Low-quality review	No financing
Frazer et al., 2021 [63]	Ireland	Evaluate the extent to which measures in LTCFs have reduced transmission of COVID-19 (SARS-CoV-2) among residents, staff, and visitors, and the effect of these implemented measures on morbidity and mortality outcomes.	Long-Term Care Facilities	Critically Low-quality review	No financing
Gonçalves et al., 2021 [64]	Portugal	Identify and evaluate pharmaceutical and/or pharmacy-based interventions in institutional long-term care settings, also mapping relevant medications.	Long-Term Care Facilities	Critically Low-quality review	No financing
Huynh et al., 2021 [65]	Canada	Answer the question: “For elderly people living in nursing homes, does the greater or lesser use of railings reduce the incidence of falls?”	Nursing homes	Critically Low-quality review	No financing
Konetzka et al., 2021 [66]	USA	Review empirical evidence on facility characteristics associated with COVID-19 cases and deaths.	Residential Aged Care	Critically Low-quality review	No financing
Li et al., 2021 [67]	USA	Provide a holistic, up-to-date perspective on nursing assistant-related factors that impact resident satisfaction in long-term care settings.	Long-Term Care Facilities	Critically Low-quality review	No financing
Wöhl et al., 2021 [68]	Germany	Update the state of knowledge on the effect of physical activity on the feasibility of activities of daily living (ADLs) for people admitted to geriatric care.	Nursing homes	Low-quality review	No financing
Chu et al., 2022 [69]	Canada	To summarize the effects of exergaming interventions on the physical, cognitive, and quality of life outcomes of elderly people (>65 years of age) living in LTCFs.	Long-Term Care Facilities	Low-quality review	Alzheimer Society of Canada New Investigator Award. This study was funded in part by grants from Drs. Chu and Biss from the New Frontiers Research Fund and the Center for Innovation in Aging and Brain Health.
Feehan et al., 2022 [70]	UK	To critically summarize the prevalence of vitamin D deficiency in nursing home residents worldwide and describe the effect of vitamin D intervention, alone or in combination with other nutrients or therapies, on improving vitamin D status and health outcomes associated with health care in nursing home residents.	Long-Term Care Facilities	Critically Low-quality review	Department of Economics. The authors thank the Rank Prize for the COVID-19 response grant.
Lijas et al., 2022 [71]	Sweden	Examine the impact of facility and staff characteristics on the risk of infectious disease outbreaks in nursing homes.	Nursing homes	Critically Low-quality review	The Swedish Research Council (Vetenskapsrådet) (2020-05850). Open access funding provided by Karolinska Institute.
Meulenbroeks et al., 2022 [72]	Australia	Fill this evidence gap by synthesizing evidence on (i) how therapy-based allied health care is delivered in RACFs globally, (ii) factors associated with levels of allied health service provision, and (iii) quality of resident care and outcomes of health care associated with allied health personnel.	Long-Term Care Facilities	Critically Low-quality review	Australian National Health and Medical Research Council (NHMRC) (APP2013953)
Narsakka et al., 2022 [73]	Finland	Synthesize evidence and provide a comprehensive understanding of environmental aspects related to physical activity in older adults in long-term care settings.	Nursing homes; Long-Term Care Facilities	Low-quality review	No financing
Albasha et al., 2023 [74]	Saudi Arabia	Synthesize the evidence on implementation strategies, implementation outcomes, and clinical outcomes included in fall prevention intervention studies.	Long-Term Care Facilities	Moderate quality review	Princess Nourah bint Abdulrahman University, Riyadh, Saudi Arabia
Kukkohov et al., 2023 [75]	Finland	Identify, critically evaluate, and synthesize evidence on the effectiveness of digital games on the physical, psychological, and social functioning and physical and social activity of elderly people in long-term care institutions.	Long-Term Care Facilities	Low-quality review	University of Oulu, including Oulu University Hospital. And Competitive State Research Financing.

* AMSTAR 2 Tool [14].

## Data Availability

This study is a systematic review and was registered on the PROSPERO platform under the number CRD42023486204.

## Supplementary Materials

The following supporting information can be downloaded at: https://www.mdpi.com/article/10.3390/geriatrics10030064/s1. Table S1: PRISMA 2020 checklist statement: an updated guideline for reporting systematic reviews, Table S2: Database search strategy: Medline via Pubmed, Embase, Web of Science, Scopus, BVS, Cochrane Library and Epistemonikos, Table S3. List of studies excluded after reading the full text (*n* = 86), Table S4. Identification of the country of long-term care facilities for the older adults and the management financing model considering the primary studies (*n* = 353) of the selected systematic reviews (*n* = 61), Table S5. Quality assessment of the systematic reviews selected in this overview (*n* = 61), Table S6. Description of the factors related to the structure and functioning adopted globally for older adults highlighted in the outcomes mobility/accessibility, technological structure, personal structure, physical structure, pharmaceutical assistance, diet/food services and risk management.
