# Supplementary material for: Overview of Systematic Reviews on Factors Related to the Structure and Functioning of Residential Long-Term Care Facilities for Older Adults
Source: Geriatrics (Basel). 2025 May 3;10(3):64. doi: 10.3390/geriatrics10030064 (PMC12101163; doi:10.3390/geriatrics10030064)
Supplement: Supplementary file 1 [file geriatrics-10-00064-s001.zip › geriatrics-3475892-supplementary.pdf]

**Supplementary File**

**Table S1-** PRISMA 2020 checklist statement: an updated guideline for reporting systematic reviews.

| Section and Topic | Item # | Checklist item | Location where item is reported |
|---|---|---|---|
| **TITLE** | | | |
| Title | 1 | Identify the report as a systematic review. | P. 1 |
| **ABSTRACT** | | | |
| Abstract | 2 | See PRISMA 2020 for the Abstracts checklist. | P. 1 |
| **INTRODUCTION** | | | |
| Rationale | 3 | Describe the rationale for the review in the context of existing knowledge. | P. 1-2 |
| Objectives | 4 | Provide an explicit statement of the objective(s) or question(s) the review addresses. | P. 2 |
| **METHODS** | | | |
| Eligibility criteria | 5 | Specify the inclusion and exclusion criteria for the review and how studies were grouped for the syntheses. | P. 3 |
| Information sources | 6 | Specify all databases, registers, websites, organizations, reference lists and other sources searched or consulted to identify studies; specify the date when each source was last searched or consulted. | P. 3 |
| Search strategy | 7 | Present the full search strategies for all databases, registers and websites, including any filters and limits used. | P. 3 |
| Selection process | 8 | Specify the methods used to decide whether the study met the inclusion criteria of the review, including how many reviewers screened each record and each report retrieved, whether they worked independently, and if applicable, details of automation tools used in the process. | P. 3 |
| Data collection process | 9 | Specify the methods used to collect data from reports, including how many reviewers collected data from each report, whether they worked independently, any processes for obtaining or confirming data from study investigators, and if applicable, details of automation tools used in the process. | P. 3 |
| Data items | 10a | List and define all outcomes for which data were sought. Specify whether all results that were compatible with each outcome domain in each study were sought (e.g., for all measures, time points, and analyses), and if not, the methods used to decide which results to collect. | P. 3 |
| | 10b | List and define all other variables for which data were sought (e.g., participant and intervention characteristics, funding sources); describe any assumptions made about any missing or unclear information. | P. 3 |
| Study risk of bias assessment | 11 | Specify the methods used to assess risk of bias in the included studies, including details of the tool(s) used, how many reviewers assessed each study and whether they worked independently, and if applicable, details of automation tools used in the process. | P. 4 |
| Effect measures | 12 | Specify for each outcome the effect measure(s) (e.g., risk ratio and mean difference) used in the synthesis or presentation of results. | NA |
| Synthesis methods | 13a | Describe the processes used to decide which studies were eligible for each synthesis (e.g. tabulating the study intervention characteristics and comparing against the planned groups for each synthesis (item #5)). | P. 3 |
| | 13b | Describe any methods required to prepare the data for presentation or synthesis, such as handling of missing summary statistics, or data conversions. | P. 2 |
| | 13c | Describe any methods used to tabulate or visually display results of individual studies and syntheses. | P. 3 |

| Section and Topic | Item # | Checklist item | Location where item is reported |
|---|---|---|---|
| | 13d | Describe any methods used to synthesize results and provide a rationale for the choice(s). If a meta-analysis was performed, describe the model(s) and/or method(s) to identify the presence and extent of statistical heterogeneity and provide the software package(s) used. | NA |
| | 13e | Describe any methods used to explore possible causes of heterogeneity among study results (e.g. subgroup analysis and meta-regression). | NA |
| | 13f | Describe any sensitivity analyses conducted to assess the robustness of the synthesized results. | NA |
| Reporting bias assessment | 14 | Describe any methods used to assess the risk of bias due to missing results in a synthesis (arising from reporting biases). | P.4 |
| Certainty assessment | 15 | Describe any methods used to assess certainty (or confidence) in the body of evidence for an outcome. | NA |
| **RESULTS** | | | |
| Study selection | 16a | Describe the results of the search and selection process, from the number of records identified in the search to the number of studies included in the review, ideally using a flow diagram. | P.4 |
| | 16b | Cite studies that might appear to meet the inclusion criteria but that were excluded, and explain why they were excluded. | P. 5 |
| Study characteristics | 17 | Cite each included study and present its characteristics. | P. 4-5 |
| Risk of bias in studies | 18 | Present assessments of risk of bias for each study included. | P. 13 |
| Results of individual studies | 19 | For all outcomes, present, for each study (a) summary statistics for each group (where appropriate) and (b) an effect estimate and its precision (e.g., confidence/credible interval), ideally using structured tables or plots. | P.5-14 |
| Results of syntheses | 20a | For each synthesis, provide a brief summary of the characteristics and risk of bias among contributing studies. | P. 13 |
| | 20b | Present results of all statistical syntheses conducted. If a meta-analysis was done, present for each the summary estimate and its precision (e.g., confidence/credible interval) and measures of statistical heterogeneity. If comparing groups, describe the direction of the effect. | NA |
| | 20c | Present results of all investigations of possible causes of heterogeneity among study results. | NA |
| | 20d | Present results of all sensitivity analyses conducted to assess the robustness of the synthesized results. | NA |
| Reporting biases | 21 | Present assessments of risk of bias due to missing results (arising from reporting biases) for each synthesis assessed. | P. 13 |
| Certainty of evidence | 22 | Present assessments of certainty (or confidence) in the body of evidence for each outcome assessed. | NA |
| **DISCUSSION** | | | |
| Discussion | 23a | Provide a general interpretation of the results in the context of other evidence. | P. 15 |
| | 23b | Discuss any limitations of the evidence included in the review. | P. 16-17 |
| | 23c | Discuss any limitations of the review processes used. | P. 17 |
| | 23d | Discusses implications of the results for practice, policy, and future research. | P. 16-17 |
| **OTHER INFORMATION** | | | |
| Registration | 24a | Provide registration information for the review, including the register name and | P. 17 |

| Section and Topic | Item # | Checklist item | Location where item is reported |
|---|---|---|---|
| and protocol | | registration number, or state that the review was not registered. | |
| | 24b | Indicate where the review protocol can be accessed, or state that the protocol was not prepared. | P. 17 |
| | 24c | Describe and explain any amendments to information provided at registration or in the protocol. | P. 17 |
| Support | 25 | Describe sources of financial or nonfinancial support for the review and the role of the funders or sponsors in the review. | P. 17 |
| Competing interests | 26 | Declare any competing interests of review authors. | P. 17 |
| Availability of data, code and other materials | 27 | Report which of the following are publicly available and where they can be found: template data collection forms; data extracted from included studies; data used for all analyses; analytical code; and any other materials used in the review. | P. 17 |

NA= Not Applicable

Source: Page MJ, McKenzie JE, Bossuyt PM, Boutron I, Hoffmann TC, Mulrow CD, et al. The PRISMA 2020 statement: an updated guideline for reporting systematic reviews. BMJ 2021;372:n71. doi: 10.1136/bmj.n71

**Table S2**. Database search strategy: Medline via Pubmed, Embase, Web of Science, Scopus, BVS, Cochrane Library and Epistemonikos.

| Databases | Strategy | Total | Total with filter |
|---|---|---|---|
| Medline via Pubmed | (Aged) OR (Elderly) OR (Health of Institutionalized Elderly) | 6.038.608 | 49.665 |
| | AND | | |
| | (Homes for the Aged) OR (Senior Housing) OR (Housing, Senior) OR (Residential Aged Care Facility) OR (Old Age Homes) OR (Home, Old Age) OR (Homes, Old Age) OR (Old Age Home) OR (Housing for the Elderly) OR (Life Care Centers, Retirement) OR (Continuing Care Retirement Centers) OR (Insurance, Long-Term Care) OR (Insurance, Long Term Care) OR (Long-Term Care Insurance) OR (Long Term Care Insurance) OR (Residential Facilities) OR (Facilities, Residential) OR (Facility, Residential) OR (Residential Facility) OR (Institutional care) OR (Residential care) | 1.140.889 | 33.038 |
| | AND | | |

| | | | |
|---|---|---|---|
| | (Health Services for the Aged) OR (Geriatric Health Services) OR (Health Services for the Elderly) OR (Health Services, Geriatric) OR (Geriatric Health Service) OR (Health Service, Geriatric) OR (Service, Geriatric Health) OR (Services, Geriatric Health) OR (Health Services for Aged) OR (Terminal Care) OR (Care, Terminal) OR (End of Life Care) OR (End-Of-Life Care) OR (Care, End-Of-Life) OR (End-Of-Life Cares) OR (Risk Management) OR (Management, Risk) OR (Management, Risks) OR (Risks Management) OR (Reporting, Hospital Incident) OR (Reportings, Hospital Incident) OR (Reportings, Hospital Risk) OR (Hospital Risk Reporting) OR (Hospital Risk Reportings) OR (Reporting, Hospital Risk) OR (Risk Reporting, Hospital) OR (Risk Reportings, Hospital) OR (Incident Reportings, Hospital) OR (Incident Reporting, Hospital) OR (Hospital Incident Reportings) OR (Voluntary Patient Safety Event Reporting) OR (Hospital Incident Reporting) OR (Incident Reporting) OR (Incident Reportings) OR (Reporting, Incident) OR (Reportings, Incident) OR (Wound Infection) OR (Infection, Wound) OR (Infections, Wound) OR (Wound Infections) OR (Pressure Ulcer) OR (Pressure Ulcers) OR (Ulcer, Pressure) OR (Ulcers, Pressure) OR (Bedsore) OR (Bedsores) OR (Pressure Sore) OR (Pressure Sores) OR (Sore, Pressure) OR (Sores, Pressure) OR (Bed Sores) OR (Bed Sore) OR (Sore, Bed) OR (Sores, Bed) OR (Decubitus Ulcer) OR (Decubitus Ulcers) OR (Ulcer, Decubitus) OR (Ulcers, Decubitus) OR (Patient Safety) OR (Patient Safeties) OR (Safeties, Patient) OR (Safety, Patient) OR (Food Handling) OR (Handling, Food) OR (Food Processing) OR (Processing, Food) OR (Diet Therapy) OR (Diet Therapies) OR (Therapy, Diet) OR (Diet Therapy, Restrictive) OR (Restrictive Diet Therapies) OR (Therapy, Restrictive Diet) OR (Restrictive Diet Therapy) OR (Restriction Diet Therapies) OR (Diet Therapies, Restriction) OR (Diet Therapy, Restriction) OR (Therapy, Restriction Diet) OR (Restriction Diet Therapy) OR (Dietary Restriction) OR (Dietary Restrictions) OR (Restriction, Dietary) OR (Dietary Modification) OR (Dietary Modifications) OR (Modification, Dietary) OR (Diet Modification) OR (Diet Modifications) OR (Modification, Diet) OR (Social Capital) OR (Capital, Social) OR (Social Infrastructure) OR (Infrastructure, Social) OR (Infrastructures, Social) OR (Social Infrastructures) OR (Built Environment) OR (Built Environments) OR (Architectural Accessibility) OR (Accessibility, Architectural) OR (Ramps) OR (Ramp) OR (Physical Barriers) OR (Barrier, Physical) OR (Barriers, Physical) OR (Physical Barrier) OR (Barriers, Architectural) OR (Architectural Barrier) OR (Architectural Barriers) OR (Barrier, Architectural) OR (Facility Access) OR (Access, Facility) OR (Accesses, Facility) OR (Facility Accesses) OR | 2.271.479 | 69.387 |

| | | | |
|---|---|---|---|
| | (Mobility Limitation) OR (Limitation, Mobility) OR (Mobility Limitations) OR (Ambulation Difficulty) OR (Ambulation Difficulties) OR (Difficulties, Ambulation) OR (Difficulty, Ambulation) OR (Difficulty Ambulation) OR (Ambulatory Difficulty) OR (Ambulatory Difficulties) OR (Difficulties, Ambulatory) OR (Difficulty Walking) OR (Walking, Difficulty) OR (Workforce) OR (Workforces) OR (Human Resources) OR (Human Resource) OR (Womanpower) OR (Womanpowers) OR (Staffing) OR (Staffings) OR (Labor Supply) OR (Labor Supplies) OR (Supply, Labor) OR (Manpower) OR (Manpowers) OR (Pharmaceutical Services) OR (Services, Pharmaceutic) OR (Services, Pharmacy) OR (Pharmaceutic Services) OR (Pharmaceutic Service) OR (Service, Pharmaceutic) OR (Services, Pharmaceutical) OR (Pharmaceutical Service) OR (Service, Pharmaceutical) OR (Pharmacy Services) OR (Pharmacy Service) OR (Service, Pharmacy) OR (Pharmaceutical Care) OR (Care, Pharmaceutical) OR (Hygiene) | | |
| | Total | 145.512 | |
| | AND | | |
| | (Systematic Review) OR (Review, Systematic) OR (Meta-Analysis) | 450.015 | 360.082 |
| | Total | 3.529 | **2.860** |
| Embase | ('aged'/exp OR 'aged patient' OR 'aged people' OR 'aged person' OR 'aged subject' OR 'elderly' OR 'elderly patient' OR 'elderly people' OR 'elderly person' OR 'elderly subject' OR 'senior citizen' OR 'senium' OR 'aged' OR 'institutionalized elderly'/exp OR 'institutionalised elderly' OR 'institutionalized elderlies' OR 'institutionalized elderly' OR 'institutionalized pensioners') | 5.840.990 | 57.361 |
| | AND | | |
| | ('home for the aged'/exp OR 'CCRC (continuing care retirement center)' OR 'continuing care retirement center' OR 'geriatric homes' OR 'home for the elderly' OR 'homes for the aged' OR 'housing for the elderly' OR 'old age home' OR 'old age homes' OR 'old people home' OR 'old people`s home' OR 'retirement center' OR 'retirement centre' OR 'retirement home' OR 'senior residence facility' OR 'home for the aged' OR 'long term care insurance'/exp OR 'insurance, long-term care' OR 'long term care insurance') | 16.162 | 83 |
| | AND | | |
| | ('elderly care'/exp OR 'elderly care' OR 'health services for the aged' OR 'old age assistance') OR ('terminal care'/exp OR 'EOL care' OR 'end-of-life care' OR 'terminal care') OR ('risk management'/exp OR 'risk management' OR 'risk planning and management' OR 'risk sharing, financial') OR ('wound infection'/exp OR 'contaminated wound' OR 'infection, wound' OR 'wound contamination' OR 'wound | 2.909.122 | 3.328 |

| | | | |
|---|---|---|---|
| | infection' OR 'wound infections' OR 'wound sepsis') OR (decubitus/exp OR 'bed sore' OR 'bedsore' OR 'decubital ulcer' OR 'decubital ulcus' OR 'decubitus' OR 'decubitus ulcer' OR 'decubitus ulceration' OR 'decubitus ulcers' OR 'decubitus ulcus' OR 'decubus ulcer' OR 'pressure injury' OR 'pressure sore' OR 'pressure ulcer' OR 'sore, pressure' OR 'ulcer, pressure' OR 'ulcus decubitus') OR ('patient safety'/exp OR 'patient safety') OR ('food handling'/exp OR 'food handling' OR 'food technology' OR 'handling, food' OR 'technology, food') OR ('diet therapy'/exp OR 'diet intervention' OR 'diet therapy' OR 'diet treatment' OR 'dietary intervention' OR 'dietary therapy' OR 'dietary treatment' OR 'nutrition therapy' OR 'nutritional therapy') OR ('social capital'/exp OR 'social capital') OR ('built environment'/exp OR 'built environment') OR ('construction work and architectural phenomena'/exp OR 'architectural accessibility' OR 'building codes' OR 'construction work and architectural phenomena' OR 'facility design and construction' OR 'location directories and signs' OR 'parking facilities') OR ('walking difficulty'/exp OR 'ambulation difficulties' OR 'ambulation difficulty' OR 'ambulatory difficulties' OR 'ambulatory difficulty' OR 'dependent ambulation' OR 'difficulty walking' OR 'mobility disabilities' OR 'mobility disability' OR 'mobility limitation' OR 'mobility limitations' OR 'walking difficulties' OR 'walking difficulty') OR (workforce/exp OR 'labor force' OR 'labour force' OR 'manpower' OR 'work force' OR 'workforce') OR ('pharmacy (shop)'/exp OR 'apothecary' OR 'chemist (shop)' OR 'chemist shop' OR 'chemist`s shop' OR 'community pharmacy' OR 'community pharmacy services' OR 'pharmaceutical service' OR 'pharmaceutical services' OR 'pharmacies' OR 'pharmacy' OR 'pharmacy (shop)' OR 'retail pharmacy') OR (hygiene/exp OR 'hygiene' OR 'hygiene care' OR 'hygiene institute' OR 'hygiene, veterinary' OR 'hygienic assessment' OR 'military hygiene' OR 'regimen sanitatis' OR 'sanitary condition' OR 'skin cleansing') | | |
| | Total | 13.746 | |
| | AND | | |
| | ('systematic review'/exp OR 'review, systematic' OR 'systematic review' OR 'meta analysis'/exp OR 'analysis, meta' OR 'meta analysis' OR 'meta-analysis' OR 'metaanalysis') | 143 | **64** |
| Web of Science | (Aged) OR (Elderly) OR (Health of Institutionalized Elderly) | 4.242.252 | 210.732 |
| | AND | | |

| | | | |
|---|---|---|---|
| | (Homes for the Aged) OR (Senior Housing) OR (Housing, Senior) OR (Residential Aged Care Facility) OR (Old Age Homes) OR (Home, Old Age) OR (Homes, Old Age) OR (Old Age Home) OR (Housing for the Elderly) OR (Insurance, Long-Term Care) OR (Insurance, Long Term Care) OR (Long-Term Care Insurance) OR (Long Term Care Insurance) OR (Residential Facilities) OR (Facilities, Residential) OR (Facility, Residential) | 121.266 | 5.593 |
| | AND | | |
| | (Health Services for the Aged) OR (Geriatric Health Services) OR (Health Services for the Elderly) OR (Health Services, Geriatric) OR (Geriatric Health Service) OR (Health Service, Geriatric) OR (Service, Geriatric Health) OR (Services, Geriatric Health) OR (Health Services for Aged) OR (Terminal Care) OR (Care, Terminal) OR (End of Life Care) OR (End-Of-Life Care) OR (Care, End-Of-Life) OR (End-Of-Life Cares) OR (Risk Management) OR (Management, Risk) OR (Management, Risks) OR (Risks Management) OR (Reporting, Hospital Incident) OR (Reportings, Hospital Incident) OR (Reportings, Hospital Risk) OR (Hospital Risk Reporting) OR (Hospital Risk Reportings) OR (Reporting, Hospital Risk) OR (Risk Reporting, Hospital) OR (Risk Reportings, Hospital) OR (Incident Reportings, Hospital) OR (Incident Reporting, Hospital) OR (Hospital Incident Reportings) OR (Voluntary Patient Safety Event Reporting) OR (Hospital Incident Reporting) OR (Incident Reporting) OR (Incident Reportings) OR (Reporting, Incident) OR (Reportings, Incident) OR (Wound Infection) OR (Infection, Wound) OR (Infections, Wound) OR (Wound Infections) OR (Pressure Ulcer) OR (Pressure Ulcers) OR (Ulcer, Pressure) OR (Ulcers, Pressure) OR (Bedsore) OR (Bedsores) OR (Pressure Sore) OR (Pressure Sores) OR (Sore, Pressure) OR (Sores, Pressure) OR (Bed Sores) OR (Bed Sore) OR (Sore, Bed) OR (Sores, Bed) OR (Decubitus Ulcer) OR (Decubitus Ulcers) OR (Ulcer, Decubitus) OR (Ulcers, Decubitus) OR (Patient Safety) OR (Patient Safeties) OR (Safeties, Patient) OR (Safety, Patient) OR (Food Handling) OR (Handling, Food) OR (Food Processing) OR (Processing, Food) OR (Diet Therapy) OR (Diet Therapies) OR (Therapy, Diet) OR (Diet Therapy, Restrictive) OR (Restrictive Diet Therapies) OR (Therapy, Restrictive Diet) OR (Restrictive Diet Therapy) OR (Restriction Diet Therapies) OR (Diet Therapies, Restriction) OR (Diet Therapy, Restriction) OR (Therapy, Restriction Diet) OR (Restriction Diet Therapy) OR (Dietary Restriction) OR (Dietary Restrictions) OR (Restriction, Dietary) OR (Dietary Modification) OR (Dietary Modifications) OR (Modification, Dietary) OR (Diet Modification) OR (Diet Modifications) OR (Modification, Diet) OR (Social Capital) | 2.650.761 | 240.007 |

| | | | |
|---|---|---|---|
| | OR (Capital, Social) OR (Social Infrastructure) OR (Infrastructure, Social) OR (Infrastructures, Social) OR (Social Infrastructures) OR (Built Environment) OR (Built Environments) OR (Architectural Accessibility) OR (Accessibility, Architectural) OR (Ramps) OR (Ramp) OR (Physical Barriers) OR (Barrier, Physical) OR (Barriers, Physical) OR (Physical Barrier) OR (Barriers, Architectural) OR (Architectural Barrier) OR (Architectural Barriers) OR (Barrier, Architectural) OR (Facility Access) OR (Access, Facility) OR (Accesses, Facility) OR (Facility Accesses) OR (Mobility Limitation) OR (Limitation, Mobility) OR (Mobility Limitations) OR (Ambulation Difficulty) OR (Ambulation Difficulties) OR (Difficulties, Ambulation) OR (Difficulty, Ambulation) OR (Difficulty Ambulation) OR (Ambulatory Difficulty) OR (Ambulatory Difficulties) OR (Difficulties, Ambulatory) OR (Difficulty Walking) OR (Walking, Difficulty) OR (Workforce) OR (Workforces) OR (Human Resources) OR (Human Resource) OR (Womanpower) OR (Womanpowers) OR (Staffing) OR (Staffings) OR (Labor Supply) OR (Labor Supplies) OR (Supply, Labor) OR (Manpower) OR (Manpowers) OR (Pharmaceutical Services) OR (Services, Pharmaceutic) OR (Services, Pharmacy) OR (Pharmaceutic Services) OR (Pharmaceutic Service) OR (Service, Pharmaceutic) OR (Services, Pharmaceutical) OR (Pharmaceutical Service) OR (Service, Pharmaceutical) OR (Pharmacy Services) OR (Pharmacy Service) OR (Service, Pharmacy) OR (Pharmaceutical Care) OR (Care, Pharmaceutical) OR (Hygiene) | | |
| | Total | 38.315 | |
| | AND | | |
| | (Systematic Review) OR (Review, Systematic) OR (Meta-Analysis) | 618.284 | 331.556 |
| | Total | 1.346 | **1013** |
| Scopus | (Aged) OR (Elderly) OR (Health of Institutionalized Elderly) | 6.371.454 | 224.021 |
| | AND | | |
| | (Homes for the Aged) OR (Senior Housing) OR (Housing, Senior) OR (Residential Aged Care Facility) OR (Old Age Homes) OR (Home, Old Age) OR (Homes, Old Age) OR (Old Age Home) OR (Housing for the Elderly) OR (Life Care Centers, Retirement) OR (Continuing Care Retirement Centers) OR (Insurance, Long-Term Care) OR (Insurance, Long Term Care) OR (Long-Term Care Insurance) OR (Long Term Care Insurance) OR (Residential Facilities) OR (Facilities, Residential) OR (Facility, Residential) OR (Residential Facility) OR (Institutional care) OR (Residential care) | 298.012 | 18.091 |
| | AND | | |

| | | | |
|---|---|---|---|
| | (Health Services for the Aged) OR (Geriatric Health Services) OR (Health Services for the Elderly) OR (Health Services, Geriatric) OR (Geriatric Health Service) OR (Health Service, Geriatric) OR (Service, Geriatric Health) OR (Services, Geriatric Health) OR (Health Services for Aged) OR (Terminal Care) OR (Care, Terminal) OR (End of Life Care) OR (End-Of-Life Care) OR (Care, End-Of-Life) OR (End-Of-Life Cares) OR (Risk Management) OR (Management, Risk) OR (Management, Risks) OR (Risks Management) OR (Reporting, Hospital Incident) OR (Reportings, Hospital Incident) OR (Reportings, Hospital Risk) OR (Hospital Risk Reporting) OR (Hospital Risk Reportings) OR (Reporting, Hospital Risk) OR (Risk Reporting, Hospital) OR (Risk Reportings, Hospital) OR (Incident Reportings, Hospital) OR (Incident Reporting, Hospital) OR (Hospital Incident Reportings) OR (Voluntary Patient Safety Event Reporting) OR (Hospital Incident Reporting) OR (Incident Reporting) OR (Incident Reportings) OR (Reporting, Incident) OR (Reportings, Incident) OR (Wound Infection) OR (Infection, Wound) OR (Infections, Wound) OR (Wound Infections) OR (Pressure Ulcer) OR (Pressure Ulcers) OR (Ulcer, Pressure) OR (Ulcers, Pressure) OR (Bedsore) OR (Bedsores) OR (Pressure Sore) OR (Pressure Sores) OR (Sore, Pressure) OR (Sores, Pressure) OR (Bed Sores) OR (Bed Sore) OR (Sore, Bed) OR (Sores, Bed) OR (Decubitus Ulcer) OR (Decubitus Ulcers) OR (Ulcer, Decubitus) OR (Ulcers, Decubitus) OR (Patient Safety) OR (Patient Safeties) OR (Safeties, Patient) OR (Safety, Patient) OR (Food Handling) OR (Handling, Food) OR (Food Processing) OR (Processing, Food) OR (Diet Therapy) OR (Diet Therapies) OR (Therapy, Diet) OR (Diet Therapy, Restrictive) OR (Restrictive Diet Therapies) OR (Therapy, Restrictive Diet) OR (Restrictive Diet Therapy) OR (Restriction Diet Therapies) OR (Diet Therapies, Restriction) OR (Diet Therapy, Restriction) OR (Therapy, Restriction Diet) OR (Restriction Diet Therapy) OR (Dietary Restriction) OR (Dietary Restrictions) OR (Restriction, Dietary) OR (Dietary Modification) OR (Dietary Modifications) OR (Modification, Dietary) OR (Diet Modification) OR (Diet Modifications) OR (Modification, Diet) OR (Social Capital) OR (Capital, Social) OR (Social Infrastructure) OR (Infrastructure, Social) OR (Infrastructures, Social) OR (Social Infrastructures) OR (Built Environment) OR (Built Environments) OR (Architectural Accessibility) OR (Accessibility, Architectural) OR (Ramps) OR (Ramp) OR (Physical Barriers) OR (Barrier, Physical) OR (Barriers, Physical) OR (Physical Barrier) OR (Barriers, Architectural) OR (Architectural Barrier) OR (Architectural Barriers) OR (Barrier, Architectural) OR (Facility Access) OR (Access, Facility) OR (Accesses, Facility) OR (Facility Accesses) OR | 3.731.761 | 456.004 |

| | | | |
|---|---|---|---|
| | (Mobility Limitation) OR (Limitation, Mobility) OR (Mobility Limitations) OR (Ambulation Difficulty) OR (Ambulation Difficulties) OR (Difficulties, Ambulation) OR (Difficulty, Ambulation) OR (Difficulty Ambulation) OR (Ambulatory Difficulty) OR (Ambulatory Difficulties) OR (Difficulties, Ambulatory) OR (Difficulty Walking) OR (Walking, Difficulty) OR (Workforce) OR (Workforces) OR (Human Resources) OR (Human Resource) OR (Womanpower) OR (Womanpowers) OR (Staffing) OR (Staffings) OR (Labor Supply) OR (Labor Supplies) OR (Supply, Labor) OR (Manpower) OR (Manpowers) OR (Pharmaceutical Services) OR (Services, Pharmaceutic) OR (Services, Pharmacy) OR (Pharmaceutic Services) OR (Pharmaceutic Service) OR (Service, Pharmaceutic) OR (Services, Pharmaceutical) OR (Pharmaceutical Service) OR (Service, Pharmaceutical) OR (Pharmacy Services) OR (Pharmacy Service) OR (Service, Pharmacy) OR (Pharmaceutical Care) OR (Care, Pharmaceutical) OR (Hygiene) | | |
| | Total | 80.532 | |
| | AND | | |
| | (Systematic Review) OR (Review, Systematic) OR (Meta-Analysis) | 770.450 | 359.307 |
| | Total | 2.033 | **1.114** |
| BVS | (Aged) OR (Elderly) OR (Health of Institutionalized Elderly) OR (Idoso) OR (Idosos) OR (Pessoa de Idade) OR (Pessoa Idosa) OR (Pessoas de Idade) OR (Pessoas Idosas) OR (População Idosa) OR (Anciano) OR (Adulto Mayor) OR (Ancianos) OR (Persona de Edad) OR (Persona Mayor) OR (Personas de Edad) OR (Personas Mayores) OR (Sujet âgé) OR (Adulte âgé) OR (Adulte de 65 à 79 ans) OR (Personne âgée) OR (Personne du troisième âge) OR (Saúde do Idoso Institucionalizado) OR (Salud del Anciano Institucionalizado) OR (estado de salud del anciano institucionalizado) OR (Santé des Anciens Institutionnalisés) | 7.295.634 | 102.026 |
| | AND | | |

| | | | |
|---|---|---|---|
| | (Homes for the Aged) OR (Senior Housing) OR (Housing, Senior) OR (Residential Aged Care Facility) OR (Old Age Homes) OR (Home, Old Age) OR (Homes, Old Age) OR (Old Age Home) OR (Housing for the Elderly) OR (Life Care Centers, Retirement) OR (Continuing Care Retirement Centers) OR (Instituição de Longa Permanência para Idosos) OR (Ancianatos) OR (Asilo para Idosos) OR (Asilos para Idosos) OR (Casas de Repouso para Idosos) OR (Centro Residencial de Cuidados com Idosos) OR (ILPI) OR (Instalações Residenciais de Cuidados com Idosos) OR (Instalações Residenciais de Cuidados para Idosos) OR (Instituição Asilar) OR (Instituições Geriátricas de Longa Permanência) OR (Moradia Sênior) OR (Hogares para Ancianos) OR (asilos de ancianos) OR (casas de ancianos) OR (centro residencial de cuidados para anciano) OR (centros de larga estancia) OR (centros geriátricos de atención a largo plazo) OR (instituciones geriátricas de estancia prolongada) OR (instituciones para ancianos de larga estancia) OR (residencias de mayores) OR (residencias geriátricas para largas estancias) OR (residencias para ancianos) OR (Maisons de retraite médicalisées) OR (Insurance, Long-Term Care) OR (Insurance, Long Term Care) OR (Long-Term Care Insurance) OR (Long Term Care Insurance) OR (Residential Facilities) OR (Facilities, Residential) OR (Facility, Residential) OR (Residential Facility) OR (Institutional care) OR (Residential care) OR (Seguro de Assistência de Longo Prazo) OR (Seguro de Assistência de Longa Duração) OR (Seguro de Cuidados a Largo Plazo) OR (Assurance soins de longue durée) | 241.840 | 6.933 |
| | AND | | |
| | (Health Services for the Aged) OR (Geriatric Health Services) OR (Health Services for the Elderly) OR (Health Services, Geriatric) OR (Geriatric Health Service) OR (Health Service, Geriatric) OR (Service, Geriatric Health) OR (Services, Geriatric Health) OR (Health Services for Aged) OR (Terminal Care) OR (Care, Terminal) OR (End of Life Care) OR (End-Of-Life Care) OR (Care, End-Of-Life) OR (End-Of-Life Cares) OR OR (Serviços de Saúde para Idosos) OR (Assistência Social para Idoso) OR (Assistência à Saúde do Idoso) OR (Atenção à Saúde do Idoso) OR (Programa de Atenção à Saúde do Idoso) OR (Rede de Assistência à Saúde do Idoso) OR (Serviços Geriátricos de Saúde) OR (Servicios de Salud para Ancianos) OR (servicios de geriatría) OR (servicios de salud geriátrica) OR (servicios de salud geriátricos) OR (Services de santé pour personnes âgées) OR (Assistência Terminal) OR (Cuidados de Fim de Vida) OR (Cuidado Terminal) OR (cuidados terminales) OR (Risk Management) OR (Management, Risk) OR (Management, Risks) OR (Risks Management) OR (Reporting, Hospital Incident) OR (Reportings, Hospital | 13.462708 | 418.803 |

| | | | |
|---|---|---|---|
| | Incident) OR (Reportings, Hospital Risk) OR (Hospital Risk Reporting) OR (Hospital Risk Reportings) OR (Reporting, Hospital Risk) OR (Risk Reporting, Hospital) OR (Risk Reportings, Hospital) OR (Incident Reportings, Hospital) OR (Incident Reporting, Hospital) OR (Hospital Incident Reportings) OR (Voluntary Patient Safety Event Reporting) OR (Hospital Incident Reporting) OR (Incident Reporting) OR (Incident Reportings) OR (Reporting, Incident) OR (Reportings, Incident) OR (Gestão de Riscos) OR (Controle de Risco) OR (Controle de Riscos) OR (Controle do Risco) OR (Gerenciamento de Risco) OR (Gerenciamento de Risco de Acidentes) OR (Gestão do Risco) OR (Informação sobre Acidentes Hospitalares) OR (Gestión de Riesgos) OR (informe de incidentes hospitalários) OR (Gestion du risque) OR (Wound Infection) OR (Infection, Wound) OR (Infections, Wound) OR (Wound Infections) OR (Infecção dos Ferimentos) OR (Infecção da Ferida) OR (Infecção das Feridas) OR (Infecção de Ferida) OR (Infecção de Feridas) OR (Infecção de Ferimento) OR (Infecção de Ferimentos) OR (Infecção do Ferimento) OR (Infección de Heridas) OR (Infection de plaie) OR (Blessure infectée) OR (Infection d'une plaie) OR (Infection de blessure) OR (Plaie infectée) OR (Pressure Ulcer) OR (Pressure Ulcers) OR (Ulcer, Pressure) OR (Ulcers, Pressure) OR (Bedsore) OR (Bedsores) OR (Pressure Sore) OR (Pressure Sores) OR (Sore, Pressure) OR (Sores, Pressure) OR (Bed Sores) OR (Bed Sore) OR (Sore, Bed) OR (Sores, Bed) OR (Decubitus Ulcer) OR (Decubitus Ulcers) OR (Ulcer, Decubitus) OR (Ulcers, Decubitus) OR (Lesão por Pressão) OR (Escara de Decúbito) OR (Úlcera de Decúbito) OR (Úlcera de Pressão) OR (Úlcera por Pressão) OR (Úlceras por Pressão) OR (Úlcera por Presión) OR (escara) OR (llaga por presión) OR (úlcera de decúbito) OR (Escarre) OR (Patient Safety) OR (Patient Safeties) OR (Safeties, Patient) OR (Safety, Patient) OR (Segurança do Paciente) OR (Seguridad del Paciente) OR (Sécurité des patients) OR (Sécurité des patientes) OR (Sécurité du patient) OR (Food Handling) OR (Handling, Food) OR (Food Processing) OR (Processing, Food) OR (Manipulação de Alimentos) OR (Processamento de Alimentos) OR (Manipulación de Alimentos) OR (procesamiento de alimentos) OR (Manipulation des aliments) OR (Diet Therapy) OR (Diet Therapies) OR (Therapy, Diet) OR (Diet Therapy, Restrictive) OR (Restrictive Diet Therapies) OR (Therapy, Restrictive Diet) OR (Restrictive Diet Therapy) OR (Restriction Diet Therapies) OR (Diet Therapies, Restriction) OR (Diet Therapy, Restriction) OR (Therapy, Restriction Diet) OR (Restriction Diet Therapy) OR (Dietary Restriction) OR (Dietary Restrictions) OR (Restriction, Dietary) OR (Dietary Modification) OR (Dietary Modifications) OR (Modification, Dietary) OR (Diet Modification) OR (Diet | | |

| | | | |
|---|---|---|---|
| | Modifications) OR (Modification, Diet) OR (Dietoterapia) OR (Dieta Restritiva) OR (Dietas Restritivas) OR (Dietoterapia Restritiva) OR (Dietoterapia de Restrição) OR (Dietoterapias Restritivas) OR (Dietoterapias de Restrição) OR (Modificação na Dieta) OR (Restrição Alimentar) OR (dieta restrictiva) OR (dietas restrictivas) OR (dietoterapia de restricción) OR (dietoterapia restrictiva) OR (dietoterapias de restricción) OR (dietoterapias restrictivas) OR (modificación dietética) OR (restricción dietética) OR (terapia dietética) OR (terapia dietética restrictiva) OR (Diétothérapie) OR (Social Capital) OR (Capital, Social) OR (Social Infrastructure) OR (Infrastructure, Social) OR (Infrastructures, Social) OR (Social Infrastructures) OR (Capital Social) OR (capital social cognitivo) OR (capital social de redes) OR (capital social negativo) OR (infraestructura social) OR (Capital Social Cognitivo) OR (Capital Social Negativo) OR (Capital Social de Redes) OR (Built Environment) OR (Built Environments) OR (Infraestrutura Social) OR (Ambiente Construído) OR (Ambientes Construídos) OR (Entorno Construído) OR (Entornos Construídos) OR (Meio Ambiente Construído) OR (Meio Construído) OR (Entorno Construido) OR (ambiente construido) OR (ambientes construídos) OR (medio construído) OR (Cadre bâti) OR (Architectural Accessibility) OR (Accessibility, Architectural) OR (Ramps) OR (Ramp) OR (Physical Barriers) OR (Barrier, Physical) OR (Barriers, Physical) OR (Physical Barrier) OR (Barriers, Architectural) OR (Architectural Barrier) OR (Architectural Barriers) OR (Barrier, Architectural) OR (Facility Access) OR (Access, Facility) OR (Accesses, Facility) OR (Facility Accesses) OR (Acessibilidade Arquitetônica) OR (Acessibilidade Física) OR (Acesso a Instalações) OR (Barreiras Arquitetônicas) OR (Barreiras Físicas) OR (Estruturas de Acesso) OR (Rampas) OR (Accesibilidad Arquitectónica) OR (acceso a instalaciones) OR (barreras arquitectónicas) OR (rampas) OR (Accessibilité architecturale) OR (Mobility Limitation) OR (Limitation, Mobility) OR (Mobility Limitations) OR (Ambulation Difficulty) OR (Ambulation Difficulties) OR (Difficulties, Ambulation) OR (Difficulty, Ambulation) OR (Difficulty Ambulation) OR (Ambulatory Difficulty) OR (Ambulatory Difficulties) OR (Difficulties, Ambulatory) OR (Difficulty Walking) OR (Walking, Difficulty) OR (Limitação da Mobilidade) OR (Dificuldade da Ambulação) OR (Dificuldade da Deambulação) OR (Dificuldade de Ambulação) OR (Dificuldade de Deambulação) OR (Limitação de Mobilidade) OR (Limitación de la Movilidad) OR (dificultad de la deambulación) OR (Mobilité réduite) OR (Workforce) OR (Workforces) OR (Human Resources) OR (Human Resource) OR (Womanpower) OR (Womanpowers) OR (Staffing) OR (Staffings) OR (Labor | | |

| | | | |
|---|---|---|---|
| | Supply) OR (Labor Supplies) OR (Supply, Labor) OR (Manpower) OR (Manpowers) OR (Recursos Humanos) OR (Equipe de Trabalho) OR (Força de Trabalho) OR (Força Laboral) OR (Mão de Obra) OR (Mão de Obra Feminina) OR (Mão de Obra Masculina) OR (Mão-de-Obra) OR (Mão-de-Obra Feminina) OR (Mão-de-Obra Masculina) OR (Oferta de Trabalho) OR (Recursos Humanos) OR (Fuerza de Trabajo) OR (Fuerza Laboral) OR (Fuerza Laboral Femenina) OR (Fuerza Laboral Masculina) OR (Mano de Obra) OR (Mano de Obra Femenina) OR (Mano de Obra Masculina) OR (Effectif) OR (Main-d'oeuvre) OR (Ressources humaines) OR (Pharmaceutical Services) OR (Services, Pharmaceutic) OR (Services, Pharmacy) OR (Pharmaceutic Services) OR (Pharmaceutic Service) OR (Service, Pharmaceutic) OR (Services, Pharmaceutical) OR (Pharmaceutical Service) OR (Service, Pharmaceutical) OR (Pharmacy Services) OR (Pharmacy Service) OR (Service, Pharmacy) OR (Pharmaceutical Care) OR (Care, Pharmaceutical) OR (Assistência Farmacêutica) OR (Atenção Farmacêutica) OR (Cuidados Farmacêuticos) OR (Serviços de Assistência Farmacêutica) OR (Servicios Farmacéuticos) OR (Atención Farmacéutica) OR (Cuidados Farmacéuticos) OR (Services pharmaceutiques) OR (Services des pharmaciens) OR (Services en pharmacie) OR (Soins pharmaceutiques) OR (Suivi pharmaceutique) OR (Hygiene) OR (Higiene) OR (Higiene Pessoal) OR (Hygiène) | | |
| | Total | 118.968 | |
| | AND | | |
| | (Systematic Review) OR (Review, Systematic) OR (Meta-Analysis) OR (Revisão Sistemática) OR (Revisión Sistemática) OR (Revue systématique) OR (Metanálise) OR (Metanálises) OR (Metaanálisis) OR (Méta-analyse) | 3.321 | **1.792** |
| Cochrane Library | #1 MeSH descriptor: [Aged] in all MeSH products | 255705 | NA |
| | #2 MeSH descriptor: [Homes for the Aged] in all MeSH products | 741 | NA |
| | #3 MeSH descriptor: [Insurance, Long-Term Care] in all MeSH products | 9 | NA |
| | #4 MeSH descriptor: [Health Services for the Aged] in all MeSH products | 537 | NA |
| | #5 MeSH descriptor: [Terminal Care Health Services for the Aged] in all MeSH products | 702 | NA |
| | #6 MeSH descriptor: [Risk Management] in all MeSH products | 14026 | NA |
| | #7 MeSH descriptor: [Wound Infection] in all MeSH products | 4943 | NA |
| | #8 MeSH descriptor: [Pressure Ulcer] in all MeSH products | 963 | NA |

| | | | |
|---|---|---|---|
| | #9 MeSH descriptor: [Patient Safety] in all MeSH products | 1530 | NA |
| | #10 MeSH descriptor: [Food Handling] in all MeSH products | 988 | NA |
| | #11 MeSH descriptor: [Diet Therapy] in all MeSH products | 7770 | NA |
| | #12 MeSH descriptor: [Social Capital] in all MeSH products | 34 | NA |
| | #13 MeSH descriptor: [Architectural Acessibility] in all MeSH products | 15 | NA |
| | #14 MeSH descriptor: [Mobility Limitation] in all MeSH products | 612 | NA |
| | #15 MeSH descriptor: [Workforce] in all MeSH products | 527 | NA |
| | #16 MeSH descriptor: [Pharmaceutical Services] in all MeSH products | 2616 | NA |
| | #17 MeSH descriptor: [Hygiene] in all MeSH products | 3146 | NA |
| | #2 OR #3 | 750 | NA |
| | #4 OR #5 OR #9 OR #10 OR #11 OR #12 OR #13 OR #14 OR #15 OR #16 OR #17 OR #18 OR #19 OR #20 | 37453 | NA |
| | #1 AND #6 AND #21 | 112 | NA |
| | Filter your results Cochrane Reviews | 3 | NA |
| Epistemonikos | (Aged) OR (Elderly) OR (Health of Institutionalized Elderly) | 847.092 | 82.356 |
| | AND | | |
| | (Homes for the Aged) OR (Senior Housing) OR (Housing, Senior) OR (Residential Aged Care Facility) OR (Old Age Homes) OR (Home, Old Age) OR (Homes, Old Age) OR (Old Age Home) OR (Housing for the Elderly) OR (Life Care Centers, Retirement) OR (Continuing Care Retirement Centers) OR (Insurance, Long-Term Care) OR (Insurance, Long Term Care) OR (Long-Term Care Insurance) OR (Long Term Care Insurance) OR (Residential Facilities) OR (Facilities, Residential) OR (Facility, Residential) OR (Residential Facility) OR (Institutional care) OR (Residential care) | 65.392 | 7.965 |
| | AND | | |
| | (Health Services for the Aged) OR (Terminal Care) OR (Risk Management) OR (Wound Infection) OR (Pressure Ulcer) OR (Patient Safety) OR (Food Handling) OR (Diet Therapy) OR (Social Capital) OR (Architectural Accessibility) OR (Mobility Limitation) OR (Workforce) OR (Pharmaceutical Services) OR (Hygiene) | 333.134 | 71.095 |
| | AND | | |
| | (Systematic Review) OR (Review, Systematic) OR (Meta-Analysis) | 454.339 | 410.377 |

| | Total | 75 | 70 |
|---|---|---|---|

Source: Authors' elaboration

**Table S3.** List of studies excluded after reading the full text (n=86).

| **Does not address health requirements or models (n=58)** |
|---|
| Comondore VR, Devereaux PJ, Zhou Q, Stone SB, Busse JW, Ravindran NC, Burns KE, Haines T, Stringer B, Cook DJ, Walter SD, Sullivan T, Berwanger O, Bhandari M, Banglawala S, Lavis JN, Petrisor B, Schünemann H, Walsh K, Bhatnagar N, Guyatt GH. Quality of care in for-profit and not-for-profit nursing homes: systematic review and meta-analysis. BMJ. 2009 Aug 4;339:b2732. doi: 10.1136/bmj.b2732. |
| Hodgkinson B, Nay R, Wilson J. A systematic review of topical skin care in aged care facilities. J Clin Nurs. 2007 Jan;16(1):129-36. doi: 10.1111/j.1365-2702.2006.01723.x. |
| Huang P, Luo K, Wang C, Guo D, Wang S, Jiang Y, Huang W, Zhang W, Ding M, Wang J. Urinary Incontinence Is Associated With Increased All-Cause Mortality in Older Nursing Home Residents: A Meta-Analysis. J Nurs Scholarsh. 2021 Sep;53(5):561-567. doi: 10.1111/jnu.12671. Epub 2021 May 22. |
| Noha Ferrah, Briony J. Murphy, Joseph E. Ibrahim, Lyndal C. Bugeja, Margaret Winbolt, Dina LoGiudice, Leon Flicker, David L. Ranson, Resident-to-resident physical aggression leading to injury in nursing homes: a systematic review, *Age and Ageing*, Volume 44, Issue 3, May 2015, Pages 356–364,https://doi.org/10.1093/ageing/afv004 |
| Caçador C, Teixeira-Lemos E, Martins SO, Ramos F. The Role of Nutritional Status on Polypharmacy, Cognition, and Functional Capacity of Institutionalized Elderly: A Systematic Review. Nutrients. 2021 Sep 30;13(10):3477. doi: 10.3390/nu13103477. |
| Griebling, T. (2013). *Re: Systematic Review of Care Intervention Studies for the Management of Incontinence and Promotion of Continence in Older People in Care Homes with Urinary Incontinence as the Primary Focus (1966–2010). The Journal of Urology, 189(6), 2209–2210.* doi:10.1016/j.juro.2013.03.014 |
| Singh S, Degeling C, Fernandez D, Montgomery A, Caputi P, Deane FP. How do aged-care staff feel about antimicrobial stewardship? A systematic review of staff attitudes in long-term residential aged-care. Antimicrob Resist Infect Control. 2022 Jun 28;11(1):92. doi: 10.1186/s13756-022-01128-5. |
| Lee SJ, Kim MS, Jung YJ, Chang SO. The Effectiveness of Function-Focused Care Interventions in Nursing Homes: A Systematic Review. J Nurs Res. 2019 Feb;27(1):1-13. doi: 10.1097/jnr.0000000000000268. |
| Namasivayam AM, Steele CM. Malnutrition and Dysphagia in long-term care: a systematic review. J Nutr Gerontol Geriatr. 2015;34(1):1-21. doi: 10.1080/21551197.2014.1002656. |

| |
|---|
| Hutchinson AM, Milke DL, Maisey S, Johnson C, Squires JE, Teare G, Estabrooks CA. The Resident Assessment Instrument-Minimum Data Set 2.0 quality indicators: a systematic review. BMC Health Serv Res. 2010 Jun 16;10:166. doi: 10.1186/1472-6963-10-166. |
| Aspden, Trefor & Bradshaw, Siobhan & Playford, Edith & Riazi, Afsane. (2014). Quality-of-life measures for use within care homes: A systematic review of their measurement properties. Age and ageing. 43. 10.1093/ageing/afu089. |
| Dörks M, Allers K, Hoffmann F. Pro Re Nata Drug Use in Nursing Home Residents: A Systematic Review. J Am Med Dir Assoc. 2019 Mar;20(3):287-293.e7. doi: 10.1016/j.jamda.2018.10.024. Epub 2018 Dec 11. |
| Gleeson A, Noble S, Mann M. Advance care planning for home health staff: a systematic review. BMJ Support Palliat Care. 2021 Jun;11(2):209-216. doi: 10.1136/bmjspcare-2018-001680. Epub 2019 Feb 26. PMID: 30808626. |
| Davies SL, Goodman C, Bunn F, Victor C, Dickinson A, Iliffe S, Gage H, Martin W, Froggatt K. A systematic review of integrated working between care homes and health care services. BMC Health Serv Res. 2011 Nov 24;11:320. doi: 10.1186/1472-6963-11-320. |
| Sterke CS, Verhagen AP, van Beeck EF, van der Cammen TJ. The influence of drug use on fall incidents among nursing home residents: a systematic review. Int Psychogeriatr. 2008 Oct;20(5):890-910. doi: 10.1017/S104161020800714X. Epub 2008 Apr 17. |
| Roe, B., Flanagan, L., Jack, B., Barrett, J., Chung, A., Shaw, C. and Williams, K. (2011), Systematic review of the management of incontinence and promotion of continence in older people in care homes: descriptive studies with urinary incontinence as primary focus. Journal of Advanced Nursing, 67: 228-250. https://doi.org/10.1111/j.1365-2648.2010.05481.x |
| Pham T, Bugeja L, Holmes A, Ibrahim JE. Systematic Review of Randomized Controlled Trials in Australian Nursing Homes from 2000 to 2018. J Am Geriatr Soc. 2021 Apr;69(4):1086-1093. doi: 10.1111/jgs.16944. Epub 2020 Nov 20. |
| Robert S. Beardsley, David B. Larson, John S. Lyons, Gary L. Gottlieb, Peter Rabins, Barry Rovner, Health Services Research in Nursing Homes: A Systematic Review of Three Clinical Geriatric Journals, *Journal of Gerontology*, Volume 44, Issue 1, January 1989, Pages M30–M35, https://doi.org/10.1093/geronj/44.1.M30 |
| Kim DE, Kim H1, Hyun J, Lee H, Sung H, Bae D, Tak SH, Park Y, Yoon JY. Interventions Using Technologies for Older Adults in Long-term Care Facilities: A Systematic Review. Journal of Korean Academy of Community Health Nursing 2018;29(2):170-183. DOI: https://doi.org/10.12799/jkachn.2018.29.2.170 |
| Baldwin R, Chenoweth L, Dela Rama M, Wang AY. Does size matter in aged care facilities? A literature review of the relationship between the number of facility beds and quality. Health Care Manage Rev. 2017 Oct/Dec;42(4):315-327. doi: 10.1097/HMR.0000000000000116. |

| |
|---|
| Aloisio LD, Coughlin M, Squires JE. Individual and organizational factors of nurses' job satisfaction in long-term care: A systematic review. Int J Nurs Stud. 2021 Nov;123:104073. doi: 10.1016/j.ijnurstu.2021.104073. Epub 2021 Aug 26. |
| Graverholt B, Forsetlund L, Jamtvedt G. Reducing hospital admissions from nursing homes: a systematic review. BMC Health Serv Res. 2014 Jan 24;14:36. doi: 10.1186/1472-6963-14-36. |
| Hasanpour AH, Sepidarkish M, Mollalo A, Ardekani A, Almukhtar M, Mechaal A, Hosseini SR, Bayani M, Javanian M, Rostami A. The global prevalence of methicillin-resistant Staphylococcus aureus colonization in residents of elderly care centers: a systematic review and meta-analysis. Antimicrob Resist Infect Control. 2023 Jan 29;12(1):4. doi: 10.1186/s13756-023-01210-6. |
| Jokanovic N, Tan EC, Dooley MJ, Kirkpatrick CM, Bell JS. Prevalence and factors associated with polypharmacy in long-term care facilities: a systematic review. J Am Med Dir Assoc. 2015 Jun 1;16(6):535.e1-12. doi: 10.1016/j.jamda.2015.03.003. Epub 2015 Apr 11. |
| Santos BP, Andrade MJC, Silva RO, Menezes E da C. Dysphagia in the elderly in long-stay institutions - a systematic literature review. Rev CEFAC [Internet]. 2018Jan;20(1):123–30. Available from: https://doi.org/10.1590/1982-021620182013817 |
| Yong B, Lin R, Xiao H. Factors associated with nursing home adjustment in older adults: A systematic review. Int J Nurs Stud. 2021 Jan;113:103790. doi: 10.1016/j.ijnurstu.2020.103790. Epub 2020 Oct 14. |
| Palese A, Menegazzi G, Tullio A, Zigotti Fuso M, Hayter M, Watson R. Functional Decline in Residents Living in Nursing Homes: A Systematic Review of the Literature. J Am Med Dir Assoc. 2016 Aug 1;17(8):694-705. doi: 10.1016/j.jamda.2016.04.002. Epub 2016 May 24. |
| Jain B, Syed S, Hafford-Letchfield T, O'Farrell-Pearce S. Dog-assisted interventions and outcomes for older adults in residential long-term care facilities: A systematic review and meta-analysis. Int J Older People Nurs. 2020 Sep;15(3):e12320. doi: 10.1111/opn.12320. Epub 2020 May 11. |
| Childs A, Zullo AR, Joyce NR, McConeghy KW, van Aalst R, Moyo P, Bosco E, Mor V, Gravenstein S. The burden of respiratory infections among older adults in long-term care: a systematic review. BMC Geriatr. 2019 Aug 5;19(1):210. doi: 10.1186/s12877-019-1236-6. PMID: 31382895; |
| Batchelor F, Hwang K, Haralambous B, Fearn M, Mackell P, Nolte L, Detering K. Facilitators and barriers to advance care planning implementation in Australian aged care settings: A systematic review and thematic analysis. Australas J Ageing. 2019 Sep;38(3):173-181. doi: 10.1111/ajag.12639. Epub 2019 Mar 14. |

| |
| --- |
| Matus-López M. Tendencias en las políticas de atención a la dependencia de ancianos y sus reformas. Cad Saúde Pública [Internet]. 2015Dec;31(12):2475–81. Available from: https://doi.org/10.1590/0102-311X00039315 |
| Xu D, Kane RL, Shamliyan TA. Effect of nursing home characteristics on residents' quality of life: a systematic review. Arch Gerontol Geriatr. 2013 Sep-Oct;57(2):127-42. doi: 10.1016/j.archger.2013.03.015. Epub 2013 Apr 25. |
| Sadowski CA, Charrois TL, Sehn E, Chatterley T, Kim S. The role and impact of the pharmacist in long-term care settings: A systematic review. J Am Pharm Assoc (2003). 2020 May-Jun;60(3):516-524.e2. doi: 10.1016/j.japh.2019.11.014. Epub 2020 Jan 25. |
| Rahman MM, Rosenberg M, Flores G, Parsell N, Akter S, Alam MA, Rahman MM, Edejer T. A systematic review and meta-analysis of unmet needs for healthcare and long-term care among older people. Health Econ Rev. 2022 Dec 9;12(1):60. doi: 10.1186/s13561-022-00398-4. |
| Meißner, A., Schnepp, W. Staff experiences within the implementation of computer-based nursing records in residential aged care facilities: a systematic review and synthesis of qualitative research. *BMC Med Inform Decis Mak* **14**, 54 (2014). https://doi.org/10.1186/1472-6947-14-54 |
| Verrue CL, Petrovic M, Mehuys E, Remon JP, Vander Stichele R. Pharmacists' interventions for optimization of medication use in nursing homes : a systematic review. Drugs Aging. 2009;26(1):37-49. doi: 10.2165/0002512-200926010-00003. |
| Cioltan H, Alshehri S, Howe C, Lee J, Fain M, Eng H, Schachter K, Mohler J. Variation in use of antipsychotic medications in nursing homes in the United States: A systematic review. BMC Geriatr. 2017 Jan 26;17(1):32. doi: 10.1186/s12877-017-0428-1. |
| Tsuboi M, Momosaki R, Vakili M, Abo M. Nutritional supplementation for activities of daily living and functional ability of older people in residential facilities: A systematic review. Geriatr Gerontol Int. 2018 Feb;18(2):197-210. doi: 10.1111/ggi.13160. Epub 2017 Aug 31. |
| Abbott R, Orr N, McGill P, Whear R, Bethel A, Garside R, Stein K, Thompson-Coon J. How do "robopets" impact the health and well-being of residents in care homes? A systematic review of qualitative and quantitative evidence. Int J Older People Nurs. 2019 Sep;14(3):e12239. doi: 10.1111/opn.12239. Epub 2019 May 9. |
| Sawka AM, Boulos P, Beattie K, Thabane L, Papaioannou A, Gafni A, Cranney A, Zytaruk N, Hanley DA, Adachi JD. Do hip protectors decrease the risk of hip fracture in institutional and community-dwelling elderly? A systematic review and meta-analysis of randomized controlled trials. Osteoporos Int. 2005 Dec;16(12):1461-74. doi: 10.1007/s00198-005-1932-2. Epub 2005 Jul 1. |

| |
|---|
| Kim KA, Lee J, Kim D, Min D. Patient safety measurement tools used in nursing homes: a systematic literature review. BMC Health Serv Res. 2022 Nov 19;22(1):1376. doi: 10.1186/s12913-022-08814-5. PMID: 36403010; PMCID: PMC9675074. |
| Bueno AA. The configuration of nursing decision-making in the care management in long-term care institutions for the elderly. 2020.153f. Tese (Doutorado Enfermagem Fundamental)- Escola de Enfermagem de Ribeirão Preto, Universidade São Paulo. Ribeirão Preto, 2020. |
| Wallerstedt SM, Kindblom JM, Nylén K, Samuelsson O, Strandell A. Medication reviews for nursing home residents to reduce mortality and hospitalization: systematic review and meta-analysis. Br J Clin Pharmacol. 2014 Sep;78(3):488-97. doi: 10.1111/bcp.12351. |
| Crocker T, Young J, Forster A, Brown L, Ozer S, Greenwood DC. The effect of physical rehabilitation on activities of daily living in older residents of long-term care facilities: systematic review with meta-analysis. Age Ageing. 2013 Nov;42(6):682-8. doi: 10.1093/ageing/aft133. Epub 2013 Sep 4. |
| Alvarez KJ, Smaldone A, Larson EL. Burden of Hepatitis C Virus Infection Among Older Adults in Long-Term Care Settings: a Systematic Review of the Literature and Meta-Analysis. Curr Infect Dis Rep. 2016 Mar;18(4):13. doi: 10.1007/s11908-016-0518-9. |
| Bell CL, Tamura BK, Masaki KH, Amella EJ. Prevalence and measures of nutritional compromise among nursing home patients: weight loss, low body mass index, malnutrition, and feeding dependency, a systematic review of the literature. J Am Med Dir Assoc. 2013 Feb;14(2):94-100. doi: 10.1016/j.jamda.2012.10.012. Epub 2012 Dec 13. |
| Storms H, Marquet K, Aertgeerts B, Claes N. Prevalence of inappropriate medication use in residential long-term care facilities for the elderly: A systematic review. Eur J Gen Pract. 2017 Dec;23(1):69-77. doi: 10.1080/13814788.2017.1288211. PMID: 28271916; |
| Liao L, Xiao LD, Chen H, Wu XY, Zhao Y, Hu M, Hu H, Li H, Yang X, Feng H. Nursing home staff experiences of implementing mentorship programmes: A systematic review and qualitative meta-synthesis. J Nurs Manag. 2020 Mar;28(2):188-198. doi: 10.1111/jonm.12876. Epub 2020 Feb 3. |
| Sossen L, Bonham M, Porter J. Can fortified, nutrient-dense and enriched foods and drink-based nutrition interventions increase energy and protein intake in residential aged care residents? A systematic review with meta-analyses. Int J Nurs Stud. 2021 Dec;124:104088. doi: 10.1016/j.ijnurstu.2021.104088. Epub 2021 Sep 8. |

| |
|---|
| Diaz-Ordaz, K., Froud, R., Sheehan, B. *et al.* A systematic review of cluster randomised trials in residential facilities for older people suggests how to improve quality. *BMC Med Res Methodol* **13**, 127 (2013). https://doi.org/10.1186/1471-2288-13-127 |
| Wang X, Shen J, Chen Q. How PARO can help older people in elderly care facilities: A systematic review of RCT. Int J Nurs Knowl. 2022 Jan;33(1):29-39. doi: 10.1111/2047-3095.12327. Epub 2021 May 7. |
| Vermeerbergen L, Van Hootegem G, Benders J. A comparison of working in small-scale and large-scale nursing homes: A systematic review of quantitative and qualitative evidence. Int J Nurs Stud. 2017 Feb;67:59-70. doi: 10.1016/j.ijnurstu.2016.11.006. Epub 2016 Nov 13. |
| Paulis SJC, Everink IHJ, Halfens RJG, Lohrmann C, Schols JMGA. Prevalence and Risk Factors of Dehydration Among Nursing Home Residents: A Systematic Review. J Am Med Dir Assoc. 2018 Aug;19(8):646-657. doi: 10.1016/j.jamda.2018.05.009. Epub 2018 Jul 6. |
| Möhler R, Richter T, Köpke S, Meyer G. Interventions for preventing and reducing the use of physical restraints in long-term geriatric care. Cochrane Database Syst Rev. 2011 Feb 16;2011(2):CD007546. doi: 10.1002/14651858.CD007546.pub2. Update in: Cochrane Database Syst Rev. 2023 Jul 28;7:CD007546. |
| Chen H, Feng H, Liao L, Wu X, Zhao Y, Hu M, Li H, Hu H, Yang X. Evaluation of quality improvement intervention with nurse training in nursing homes: A systematic review. J Clin Nurs. 2020 Aug;29(15-16):2788-2800. doi: 10.1111/jocn.15289. Epub 2020 May 21. |
| Raban MZ, Gasparini C, Li L, Baysari MT, Westbrook JI. Effectiveness of interventions targeting antibiotic use in long-term aged care facilities: a systematic review and meta-analysis. BMJ Open. 2020 Jan 9;10(1):e028494. doi: 10.1136/bmjopen-2018-028494. |
| Forsetlund L, Eike MC, Gjerberg E, Vist GE. Effect of interventions to reduce potentially inappropriate use of drugs in nursing homes: a systematic review of randomised controlled trials. BMC Geriatr. 2011 Apr 17;11:16. doi: 10.1186/1471-2318-11-16. |
| Hall, A.M., Flodgren, G.M., Richmond, H.L. *et al.* Champions for improved adherence to guidelines in long-term care homes: a systematic review. *Implement Sci Commun* **2**, 85 (2021). https://doi.org/10.1186/s43058-021-00185-y |
| **Incomplete text (n=12)** |

| |
|---|
| Fischer T, Worch A, Nordheim J, Wulff IM, Gräske J, Meye S, Wolf-Ostermann K. Ambulant betreute Wohngemeinschaften für alte, pflegebedürftige Menschen - Merkmale, Entwicklung und Einflussfaktoren [Shared-housing arrangements for care-dependent older persons - characteristics, development and drivers]. Pflege. 2011 Apr;24(2):97-109. German. doi: 10.1024/1012-5302/a000105. |
| Prihatama RP, Yusuf A, Sari DW. Implementation of long-term care for older adults: A Systematic Review. J Pak Med Assoc. 2023 Feb;73(Suppl 2)(2):S175-S181. doi: 10.47391/JPMA.Ind-S2-39. |
| Alvarez-Barbosa F, Del Pozo-Cruz J, Del Pozo-Cruz B, García-Hermoso A, Alfonso-Rosa RM. Effects of Whole-Body Vibration on Functional Mobility, Balance, Gait Strength, and Quality of Life in Institutionalized Older People: A Systematic Review and Meta-Analysis of Randomized Controlled Trials. J Aging Phys Act. 2020 Apr 24;28(2):219-230. doi: 10.1123/japa.2019-0070. |
| Alcázar VR, Martín JC, Herrera-Usagre M.Revisión sistemática sobre recomendaciones de seguridad del paciente para centros sociosanitarios, Medicina clínica, Vol. 141, Nº. 9, 2013, págs. 397-405. |
| Forough AS, Wong SYM, Lau ETL, Santos JMS, Kyle GJ, Steadman KJ, Cichero JAY, Nissen LM. Nurse experiences of medication administration to people with swallowing difficulties living in aged care facilities: a systematic review of qualitative evidence. JBI Database System Rev Implement Rep. 2018 Jan;16(1):71-86. doi: 10.11124/JBISRIR-2016-003334. |
| McInerney BE, Cross AJ, Turner JP, Bell JS. Systematic Review of Psychotropic Adverse Drug Event Monitoring Tools for Use in Long-Term Care Facilities. J Am Med Dir Assoc. 2023 Jun;24(6):773-781.e5. doi: 10.1016/j.jamda.2023.03.003. Epub 2023 Apr 8. |
| Francis-Coad J, Etherton-Beer C, Burton E, Naseri C, Hill AM. Effectiveness of complex falls prevention interventions in residential aged care settings: a systematic review. JBI Database System Rev Implement Rep. 2018 Apr;16(4):973-1002. doi: 10.11124/JBISRIR-2017-003485. |
| Wong J, Pedersen J, Tennety N, DuBois L, Chiu R, Shah D, Malecki G, Wafford QE, Heinemann AW. Service-Delivery Competencies in Home and Community-Based Services Needed to Achieve Person-Centered Planning and Practices: A Systematic Review. J Appl Gerontol. 2023 Mar;42(3):493-504. doi: 10.1177/07334648221139476. Epub 2022 Nov 17. |
| Huang Y, Wei WI, Correia DF, Ma BHM, Tang A, Yeoh EK, Wong SYS, Ip M, Kwok KO. Antibiotic use for respiratory tract infections among older adults living in long-term care facilities: a systematic review and meta-analysis. J Hosp Infect. 2023 Jan;131:107-121. doi: 10.1016/j.jhin.2022.09.016. Epub 2022 Oct 4. |

| |
|---|
| Sadeq A, Strugaru M, Almutairi M, Stewart D, Ryan C, Grimes T. Interprofessional Interventions Involving Pharmacists and Targeting the Medicines Management Process Provided to Older People Residing in Nursing Homes: A Systematic Review and Meta-Analysis of Randomised Controlled Trials. Drugs Aging. 2022 Oct;39(10):773-794. doi: 10.1007/s40266-022-00978-3. Epub 2022 Oct 4. |
| Kehinde JO, Pope C, Amella EJ. Methodological issues in fall prevention research involving older adults in long-term care facilities. Res Gerontol Nurs. 2011 Oct;4(4):294-304. doi: 10.3928/19404921-20110503-02. Epub 2011 May 18. |
| Portillo CU, Calvo Arenillas JI, Miralles PM. Occupational Therapy Interventions for the Improvement of the Quality of Life of Healthy Older Adults Living in Nursing Homes: A Systematic Review. Am J Health Promot. 2023 Jun;37(5):698-704. doi: 10.1177/08901171221145159. Epub 2022 Dec 11. |
| **Non-elderly population (n=4)** |
| Armijo-Olivo S, Craig R, Corabian P, Guo B, Souri S, Tjosvold L. Nursing Staff Time and Care Quality in Long-Term Care Facilities: A Systematic Review. Gerontologist. 2020 Apr 2;60(3):e200-e217. doi: 10.1093/geront/gnz053. |
| Wendland J, Parizet L. Benefits and challenges of intergenerational child daycare and senior programs or facilities: A systematic review of the literature. Annales Médico-psychologiques, revue psychiatrique Volume 181, Issue 6, June 2023, Pages 487-494. https://doi.org/10.1016/j.amp.2022.02.020 |
| Kwok KO, Read JM, Tang A, Chen H, Riley S, Kam KM. A systematic review of transmission dynamic studies of methicillin-resistant Staphylococcus aureus in non-hospital residential facilities. BMC Infect Dis. 2018 Apr 18;18(1):188. doi: 10.1186/s12879-018-3060-6. |
| Lan SH, Lu LC, Yen YY, Hsieh YP, Chen JC, Wu WJ, Lan SJ, Lin LY. Tube Feeding among Elder in Long-Term Care Facilities: A Systematic Review and Meta-Analysis. J Nutr Health Aging. 2017;21(1):31-37. doi: 10.1007/s12603-016-0717-y. |
| **Not LTCF (n=8)** |
| Meddings J, Saint S, Krein SL, Gaies E, Reichert H, Hickner A, McNamara S, Mann JD, Mody L. Systematic Review of Interventions to Reduce Urinary Tract Infection in Nursing Home Residents. J Hosp Med. 2017 May;12(5):356-368. doi: 10.12788/jhm.2724. |
| Church J, Goodall S, Norman R, Haas M. An economic evaluation of community and residential aged care falls prevention strategies in NSW. N S W Public Health Bull. 2011 Jun;22(3-4):60-8. doi: 10.1071/NB10051. PMID: 21632001. |
| Boult C, Green AF, Boult LB, Pacala JT, Snyder C, Leff B. Successful models of comprehensive care for older adults with chronic conditions: evidence for the Institute of Medicine's "retooling for an aging America" report. J Am Geriatr Soc. 2009 Dec;57(12):2328-37. doi: 10.1111/j.1532-5415.2009.02571.x. |

| |
|---|
| Johnson S, Bacsu J, Abeykoon H, McIntosh T, Jeffery B, Novik N. No Place Like Home: A Systematic Review of Home Care for Older Adults in Canada. Can J Aging. 2018 Dec;37(4):400-419. doi: 10.1017/S0714980818000375. Epub 2018 Sep 4. |
| Manias E, Bucknall T, Hutchinson A, Dow B, Borrott N. Resident and family engagement in medication management in aged care facilities: a systematic review. Expert Opin Drug Saf. 2021 Nov;20(11):1391-1409. doi: 10.1080/14740338.2021.1935862. Epub 2021 Jun 14. |
| Li Y, Chen P, Liu C, Chien W, Tung T. The Association between Quality of Life and Nursing Home Facility for the Elderly Population: A Systematic Review and Meta-Analysis. International Journal of Gerontology 15 (2021) 16-24. https://doi.org/10.6890/IJGE.202101_15(1).0004 |
| Nyman SR, Victor CR. Older people's recruitment, sustained participation, and adherence to falls prevention interventions in institutional settings: a supplement to the Cochrane systematic review. Age Ageing. 2011 Jul;40(4):430-6. doi: 10.1093/ageing/afr016. Epub 2011 Apr 18. |
| Narsakka N, Suhonen R, Stolt M. Environment in institutional care settings as a promoting factor for older individuals' mobility: A systematic review. Scand J Caring Sci. 2022 Jun;36(2):382-392. doi: 10.1111/scs.13053. Epub 2021 Dec 11. PMID: 34893995; |
| **Specific for pathologies (n=3)** |
| Van Brunt K, Curtis B, Brooks K, Heinloth A, de Cassia Castro R. Insulin use in long term care settings for patients with type 2 diabetes mellitus: a systematic review of the literature. J Am Med Dir Assoc. 2013 Nov;14(11):809-16. doi: 10.1016/j.jamda.2013.08.018. |
| Morrison EJ, Middleton J, Lanza S, Cowen JE, Hewitt K, Walker SL, Nicholls M, Rajan-Iyer J, Fletcher J, Cassell JA. Do we know how scabies outbreaks in residential and nursing care homes for the elderly should be managed? A systematic review of interventions using a novel approach to assess evidence quality. Epidemiol Infect. 2019 Aug 5;147:e250. doi: 10.1017/S0950268819001249. PMID: 31496448; |
| Garcia TJ, Brown SA. Diabetes management in the nursing home: a systematic review of the literature. Diabetes Educ. 2011 Mar-Apr;37(2):167-87. doi: 10.1177/0145721710395330. |
| **Review protocol (n=1)** |
| Hines S, Wilson J, McCrow J, Abbey J, Sacre S. Oral liquid nutritional supplements for people with dementia in residential aged care facilities. Int J Evid Based Healthc. 2010 Dec;8(4):248-51. doi: 10.1111/j.1744-1609.2010.00186.x. |

Source: Authors' elaboration

**Table S4**. Identification of the country of long-term care facilities for the older adults and the management financing model considering the primary studies (n= 353) of the selected systematic reviews (n=61).

| **Outcome** | **Review** | **Primary Studies** | **Country of Residential Facilities** | **Management financing model** |
|---|---|---|---|---|
| Diet Food Services | Abbott et al/2013 | Appleton et al. 2008 | Northern Ireland | Not reported |
| | | Lorefalt B, Wilhelmsson S. 2012 | Sweden | Not reported |
| Personnel Structure | Albasha et al/2023 | Leverenz MD, Lape J. 2018 | USA | Not reported |
| | | Kato et al. 2008 | Japan | Not reported |
| | | Aguwa H. 2019 | Not reported | Not reported |
| Personnel Structure | Ali et al/ 2021 | Nace et al. 2020 | USA | Not reported |
| | | Ailabouni et al. 2019 | New Zealand | Not reported |
| | | Pasay et al. 2019 | Canada | Private non-profit |
| | | Hashimoto et al. 2019 | Japan | Not reported |
| | | Stuhec M, Bratoviÿ N, MrharA. 2019 | Slovenia | Not reported |
| | | Frankenthal et al. 2014 | Israel | Not reported |
| | | Kersten et al. 2012 | Norway | Not reported |
| Pharmaceutical care | Ali et al/ 2021 | Frankenthal et al. 2014 | Israel | Not reported |
| | | Hashimoto et al. 2019 | Japan | Not reported |
| | | Avorn et al. 1992 | USA | Not reported |
| | | Lapane et al. 2011 | USA | Private for profit |
| | | Zermansky et al. 2006 | UK | Not reported |
| | | Claesson CB, Schmid IK. 1998 | Sweden | Not reported |
| Pharmaceutical care | Alldred et al/ 2016 | Kane et al. 2003 | Incomplete Text | Incomplete Text |
| Personnel Structure | Barker et al/2018 | Fan et al. 2011 | Incomplete Text | Incomplete Text |
| | | Klaasen et al. 2009 | Incomplete Text | Incomplete Text |
| | | Hutchinson et al. 2015 | Australia | Private non-profit |
| | | Shapiro et al. 1987 | Canada e USA | Not reported |
| | | Connolly et al. 2016 | New Zealand | Not reported |

| | | | | |
|---|---|---|---|---|
| | | Schippinger et al. 2012 | Austria | Not reported |
| | | Lisk et al. 2012 | UK | Not reported |
| | | D'Arcy et al. 2013 | USA | Private for profit |
| | | Boorsma et al. 2011 | Netherlands | Private for profit |
| | | Tamura et al. 2011 | Hawai | Private non-profit |
| | | Monroe et al. 2011 | USA | Private for profit |
| | | Pittrow et al. 2003 | Germany | Private for profit |
| | | Gloth et al. 2011 | USA | Private for profit |
| | | McKnee et al. 2016 | Northern Ireland | Public |
| | | Kane et al. 2004 | USA | Private for profit |
| | | Codde et al. 2010 | Australia | Not reported |
| | | El-Masri et al. 2015 | Canada | Private non-profit |
| | | Fan et al. 2016 | Australia | Public |
| | | Boyd et al/2014 | New Zealand | Not reported |
| | | Street et al/2014 | Australia | Not reported |
| | | Rantz et al/2017 | USA | Private for profit |
| | | Hicks et al/2004 | Incomplete Text | Incomplete Text |
| Personnel Structure | Bostick et al/2006 | Anderson et al/1998 | USA | Private for profit |
| | | Bosticks et al/ 2004 | USA | Private for profit |
| | | Harrington et al/2000 | USA | Not reported |
| | | Harrington et al/2002 | USA | Private for profit /Private non-profit/ Public |
| | | Jutkowitz et al/2002 | USA | Not reported |
| | | Anderson et al/1997 | USA | Public |
| | | Anderson et al/2003 | USA | Private non-profit |
| | | Intrator et al/1999 | USA | Public/ Private non-profit |
| | | Spilsbury et al/2011 | Not reported | Not reported |
| | | Rantz et al/2004 | USA | Public, Private for profit/ Private non-profit |
| | | Berlowitz et al/1999 | USA | Not reported |

| | | | | |
|---|---|---|---|---|
| | | Cohen et al/1996 | USA | Public/ Private non-profit/ Private for profit |
| | | Castle & Fogel/1998 | USA | Private for profit/ Private non-profit |
| | | Asmuth MV/2004 | Incomplete Text | Incomplete Text |
| Personnel Structure | Bradshaw et al/2012 | Bergland & Kirkevold/2005 | Norway | Not reported |
| | | Cooney et al/2009 | UK | Public/ Private for profit |
| | | Edwards et al/2003 | Australia | Not reported |
| | | Hauge & Heggen/2007 | Norway | Not reported |
| | | Shuang et al/2023 | China | Not reported |
| | | Bergland & Kirkevold/2006 | Norway | Not reported |
| | | Coughlan & Ward/2007 | Canada | Not reported |
| | | Riazi et al/2012 | UK | Not reported |
| | | De Boer et al.2007 | Netherlands | Not reported |
| Personnel Structure | Brett et al/2019 | Burkett LS/2016 | Incomplete Text | Incomplete Text |
| Diet Food Services | Bunn et al/2015 | Taylor KA & Barrt SI et al. 2006 | Canada | Not reported |
| | | Robinson et al.2002 | USA | Not reported |
| Technological Structure | Bunn et al/2015 | Fries et al.1997 | USA | Public/ Private non-profit |
| Risk management | Cao et al/2018 | Beudart et al. 2013 | Belgium | Not reported |
| | | Brustio et al. 2015 | Italy | Private for profit |
| | | Buckinx et al. 2014 | Belgium | Private for profit |
| | | Kovács et al. 2013 | Hungary | Not reported |
| | | Sievanen et al. 2014 | Finland | Not reported |
| Risk management | Chan et al/2014 | Taylor et al.1992 | Incomplete Text | Incomplete Text |
| | | Monto et al.2000 | USA | Not reported |
| | | Gross et al. 1988 | USA | Not reported |
| | | Saah et al. 1986 | USA | Not reported |
| | | Saito et al. 2002 | Japan | Not reported |
| | | Murayama et al. 1999 | Japan | Not reported |

| | | | | |
|---|---|---|---|---|
| | | Strassburg et al. 1986 | USA | Not reported |
| | | Ching Chan et al. 2013 | China | Not reported |
| | | McLarin et al. 2015 | Australia | Not reported |
| Pharmaceutical care | Chen et al/ 2019 | Nishtala et al. 2009 | Australia | Not reported |
| | | Poudel et al. 2015 | Australia | Not reported |
| | | Gheewala et al. 2014 | Australia | Not reported |
| Personnel Structure | | Koria et al. 2018 | Australia | Not reported |
| Mobility-Accessibility | Chu et al/2022 | Yesilyaprak et al. 2016 | Turkey | Not reported |
| | | Delbroek, Vermeylen, Spildoorenl. 2017 | Belgium | Private for profit |
| | | Cicek,Ozdincler,Tarakci. 2020 | Turkey | Not reported |
| | | Chu et al. 2022 | Not reported | Not reported |
| | | Hsieh et al. 2018 | Taiwan | Not reported |
| | | Janssen,Tange,Arends. 2012 | Netherlands | Not reported |
| | | Ellmers et al. 2018 | UK | Not reported |
| | | Pichierri,Murer,Bruin. 2012 | Suíça | Not reported |
| | | Ogawa et al. 2019 | USA | Not reported |
| | | Valiani et al. 2017 | USA | Not reported |
| Personnel Structure | Clarkson et al/2018 | Zermansky et al. 2006 | UK | Not reported |
| | | Ryden et al.2000 | USA | Public |
| | | Furniss et al.2000 | USA | Not reported |
| Risk management | Diehl et al/2016 | Van Gaal et al.2011 | Incomplete Text | Incomplete Text |
| Personnel Structure | Donald et al/2013 | Ryden et al.1999 | Incomplete Text | Incomplete Text |
| | | Ryden et al.2000 | USA | Public |
| | | Krichbaum et al.2000 | Incomplete Text | Incomplete Text |
| | | Snyder et al.1998 | USA | Not reported |
| | | Krichbaum et al.2005 | USA | Not reported |
| | Donaldson et al/2019 | Kwok , Woo , Kwan, 2001 | Incomplete Text | Incomplete Text |

| | | | | |
|---|---|---|---|---|
| Diet Food Services | | Stange et al. 2013 | Incomplete Text | Incomplete Text |
| | | Lauque et al.2000 | France | Private for profit |
| | | Lee et al.2003 | Taiwan | Private for profit |
| | | Smoliner et al. 2008 | Germany | Not reported |
| | | Bonnefoy et al.2003 | France | Not reported |
| | | Wouters-Wesseling et al.2002 | Netherlands | Not reported |
| Personnel Structure | Dwyer et al/2011 | Hasson et al.2008 | Northern Ireland | Private for profit |
| | | Capezuti et al.2007 | USA | Private non-profit |
| Technological Structure | Edirippulige et al/2013 | Zelickson & Homan. 1997 | Incomplete Text | Incomplete Text |
| | | Corcoran, Hui, Woo. 2002 | China | Not reported |
| | | Rabinowitz et al. 2010 | USA | Not reported |
| | | Yeung et al.2009 | USA | Not reported |
| | | Shores et al. 2004 | USA | Not reported |
| | | Bratan et al. 2005 | London | Not reported |
| | | Laflamme et al.2005 | USA | Public |
| | | Clarke et al.2007 | Incomplete Text | Incomplete Text |
| Diet Food Services | Feehan et al/2022 | Delomas et al. 2016 | Incomplete Text | Incomplete Text |
| | | Rezza et al. 2018 | Incomplete Text | Incomplete Text |
| | | Schwalfenberg & Genuis 2010 | Canada | Not reported |
| | | Chel et al. 2011 | Netherlands | Not reported |
| | | Dinizulu et al.2011 | Ireland | Not reported |
| | | Shin & Minden 2011 | USA | Not reported |
| | | Ioannidis et al.2012 | Canada | Private for profit |
| | | Sambrook et al.2012 | Australia | Not reported |
| | | Tellioglua et al.2012 | Turkey | Not reported |
| | | Durvasula et al.2014 | Australia | Not reported |
| | | Feldman et al. 2014 | Canada | Not reported |
| | | Veleva, Chel, Achterberg. 2014 | Netherlands | Not reported |
| | | Wijnen et al.2015 | Netherlands | Not reported |
| | | Schwartz et al. 2016 | USA | Not reported |

| | | | | |
|---|---|---|---|---|
| | | ToreņÿWielema et al. 2018 | Netherlands | Not reported |
| | | Samefors, Tenglblad, Östrgren. 2020 | Sweden | Not reported |
| | | Okan, Zincir,Deveci. 2021 | Turkey | Not reported |
| | | Mueangpaisarn & Chaiamnuay, 2019 | Thailand | Not reported |
| Physical structure | Feehan et al/2022 | Samefors, Tenglblad, Östrgren. 2020 | Sweden | Not reported |
| Pharmaceutical care | Ferrah et al/2017 | Desai et al. 2012 | Incomplete Text | Incomplete Text |
| | | Desai et al. 2011 | USA | Not reported |
| | | Crespin et al.2010 | USA | Not reported |
| | | Greene et al. 2018 | USA | Not reported |
| | | Lane et al. 2014 | USA | Not reported |
| Personnel Structure Risk management | Flanagan et al/2013 | Hu et al. 1989 | Incomplete Text | Incomplete Text |
| | | Schenelle et al.1988 | USA | Not reported |
| | | Sowell et al.1987 | USA | Not reported |
| Risk management | Frazer et al/2021 | Arons et al. 2020 | USA | Not reported |
| | | Borras-Bermejo et al. 2020 | Spain | Public |
| | | Dora et al.2020 | USA | Not reported |
| | | Eckardt et al.2020 | USA | Not reported |
| | | Dutey-Magni et al.2021 | UK | Private for profit |
| | | Feaster & Goh. 2020 | USA | Not reported |
| | | Graham et al. 2020 | UK | Not reported |
| | | Hand et al.2018 | USA | Not reported |
| | | Kennelly et al. 2021 | Ireland | Public/ Private for profit |
| | | Kimball et al.2020 | USA | Not reported |
| | | Klein et al.2020 | Germany | Not reported |
| | | Lennon et al. 2020 | USA | Not reported |
| | | Louie et al. 2021 | USA | Not reported |
| | | McMichael et al.2020 | USA | Not reported |
| | | Office for National Statistics, 2020 | UK | Not reported |
| | | Patel et al. 2020 | USA | Not reported |

| | | | | |
|---|---|---|---|---|
| | | Roxby et al.2020 | USA | Not reported |
| | | Sacoo et al.2020 | France | Not reported |
| | | Telford et al. 2020 | USA | Not reported |
| Technological Structure | Ghavarskhar et al/2018 | Jenkens, Carder,Maher.2008 | Incomplete Text | Incomplete Text |
| | | Just, Deyoung, Van Dyk. 1995 | USA | Not reported |
| | | Wilson KB.2007 | USA | Not reported |
| | | Wink DM & Holcomb LO.2002 | Not reported | Not reported |
| | | Zimmerman S & Sloane PD.2007 | USA | Not reported |
| Physical structure | Ghavarskhar et al/2018 | Jenkens, Carder,Maher.2008 | Incomplete Text | Incomplete Text |
| | | Just, Deyoung, Van Dyk. 1995 | USA | Not reported |
| | | Wilson KB.2007 | USA | Not reported |
| | | Wink DM & Holcomb LO.2002 | Not reported | Not reported |
| | | Zimmerman S & Sloane PD.2007 | USA | Not reported |
| Personnel Structure | Gonçalves et al/2021 | Lapane et al. 2011 | USA | Private for profit/ Private non-profit |
| | | Locca et al.2008 | Switzerland | Not reported |
| | | Roberts et al. 2008 | Australia | Not reported |
| | | Lapane et al. 2011 | USA | Private for profit/ Private non-profit |
| Pharmaceutical care | Huey Lee et al/2019 | Furniss et al.2000 | USA | Not reported |
| | | Zermansky et al. 2006 | UK | Not reported |
| | | Frankenthal et al. 2014 | Israel | Not reported |
| | | Crotty et al. 2004 | Incomplete Text | Incomplete Text |
| | | Verrue et al.2002 | Incomplete Text | Incomplete Text |
| | | Thompson et al. 1984 | USA | Not reported |
| | | Lee et al. 2017 | UK | Private non-profit |
| | | Crotty et al. 2004 | Australia | Not reported |
| Personnel Structure | Huey Lee et al/2019 | Wilcher DE &,Cooper JW | Not reported | Not reported |
| | | Crotty et al. 2004 | Australia | Not reported |
| | | Wilcher DE &,Cooper JW | Not reported | Not reported |

| | | | | |
|---|---|---|---|---|
| | | Elzarian EJ, Shirachi DY, Jones | USA | Not reported |
| | | Cooper JW. 1985 | Not reported | Not reported |
| Risk management | Hughes et al/2013 | Baldwin et al. 2010 | UK | Not reported |
| Risk managements | Huynh et al/2021 | Agashivala N. & Wu WK. 2009 | Incomplete Text | Incomplete Text |
| | | Meyer et al. 2008 | Germany | Not reported |
| | | Fonad et al. 2007 | Sweden | Public |
| | | Fonad et al.2009 | Sweden | Public |
| | | Luo H, Lin M, Castle N. | USA | Not reported |
| | | Lopez-Soto et al. 2016 | Italy | Not reported |
| | | Ko¨ pke et al. 2012 | Germany | Not reported |
| | | Haynes RB. 2006 | Not reported | Not reported |
| Risk managements | Hye Lee et al/2019 | SSK Ho, MMY Tse. 2012 | China | Not reported |
| | | Mody et al. 2015 | USA | Private for profit /Private non-profit |
| | | Schweon et al. 2013 | USA | Private for profit |
| | | Eveillard et al. 2011 | France | Not reported |
| Personnel Structure | Wu et al/2020 | Morrison-Pandy et al 2015 | USA | Not reported |
| Pharmaceutical care | Vlaeyen et al/2015 | Pattterson et al 2010 | Northern Ireland | Private for profit/ Private non-profit |
| Physical structure | Van Malderen et al/2013 | Kane et al. 2007 | USA | Not reported |
| Physical structure | van den Berg et al/2019 | Bengtsson & Carlsson, 2005 | Sweden | Not reported |
| | | Raske, 2010 | USA | Not reported |
| | | Heath & Gifford, 2001 | Canada | Not reported |
| | | Rappe et al., 2006 | Finland | Not reported |
| | | Bengtsson & Carlsson, 2013 | Sweden | Not reported |
| | | Potter et al., 2018 | UK | Public/ Private for profit |

| | | | | |
|---|---|---|---|---|
| | | Rappe & Kivelä, 2005 | Finland | Not reported |
| | | Senes et al., 2012 | Italy | Not reported |
| | | Chapman et al., 2007 | USA | Not reported |
| | | Morgan & Stewart, 1999 | Canada | Not reported |
| | | Chao et al., 2014 | Taiwan | Not reported |
| | | Oguz et al., 2010 | Turkey | Not reported |
| | | Durvasula et al . , 2010 | Australia | Not reported |
| Mobility-Accessibility | Valenzuela et al/2012 | Dorner et al. 2007 | Áustria | Not reported |
| | | Fisher et al. 1991 | Incomplete Text | Incomplete Text |
| | | McMurdo et al. 1994 | Incomplete Text | Incomplete Text |
| Pharmaceutical care | Thiruchelvam et al/2016 | Milos et al. 2013 | Sweden | Public/ Private for profit |
| | | Alldred et al 2007 | UK | Not reported |
| | | Baqir et al 2014 | UK | Not reported |
| Pharmaceutical care | Tamura et al/2012 | Dwyer et al 2010 | USA | Private for profit |
| Diet Food Services | Sinclair et al/2011 | Sinclair et al/2011 | UK | Not reported |
| Mobility-Accessibility | Silva et al/2013 | Becker et al 2003 | Germany | Not reported |
| | | Dyer et al 2004 | England | Not reported |
| | | Faber et al 2006 | Incomplete Text | Incomplete Text |
| | | McMurdo et al. 2000 | Scotland | Not reported |
| | | Rosendahl et al. 2008 | Sweden | Not reported |
| | | Sihvonen et al 2004 | Not reported | Not reported |
| | | Nowalk et al 2001 | Not reported | Not reported |
| | | Schnelle et al 2003 | USA | Private for profit |
| Risk managements | Senderovich et al/2019 | Joesoef et al 2012 | USA | Private for profit |
| Risk managements | Schoberer et al/2019 | Buckinx et al 2014 | Belgium | Not reported |
| | | Cadore et al 2014 | Spain | Not reported |
| | | DeSure et al 2013 | USA | Not reported |

| | | | | |
|---|---|---|---|---|
| | | Faber et al 2006 | Incomplete Text | Incomplete Text |
| | | Fu et al 2015 | China | Not reported |
| | | Kerse et al 2008 | New Zealand | Not reported |
| | | Kovacs et al 2012 | Hungary | Not reported |
| | | Kovacs et al 2013 | Hungary | Not reported |
| | | Lord et al 2003 | Australia | Not reported |
| | | Mulrow et al 1994 | USA | Private for profit |
| | | Nowalk et al 2001 | Not reported | Not reported |
| | | Rolland et al 2007 | France | Not reported |
| | | Rosendahl et al. 2008 | Sweden | Not reported |
| | | Sakamoto et al. 2006 | Japan | Not reported |
| | | Schnelle et al 2003 | USA | Private for profit |
| | | Schoenfelder et al 2000 | Incomplete Text | Incomplete Text |
| | | Shimada et al 2004 | Japan | Public |
| | | Sihvonen et al 2004 | Finland | Not reported |
| | | Wolf 2003 | USA | Private for profit |
| Risk managements | Sawka et al/2006 | Spiegelhalter et al 2004 | Incomplete Text | Incomplete Text |
| Pharmaceutical care | Reyes-Alcázar et al/2013 | Department of Health and Aged Care | Australia | Not reported |
| Technological Structure | Panza et al/2018 | Van Craen et al 2010 | Not reported | Not reported |
| Personnel Structure | | Boyd et al. 2014 | New Zealand | Private for profit |
| Diet Food Services | | Morley et al 2016 | USA | Private for profit |
| Risk managements | Pagan et al/2015 | Baier et al 2003 | Incomplete Text | Incomplete Text |
| | | Shannon et al 2012 | USA | Private for profit |
| Personnel Structure | Nguyen et al/2019 | Fleet et al 2014 | UK | Not reported |

| | | | | |
|---|---|---|---|---|
| Personnel Structure | Neyens et al/2011 | Zermansky et al 2006 | UK | Not reported |
| Diet Food Services | | Bischoff et al 2003 | Switzerland | Not reported |
| | | Bischoff-Ferrari et al 2006 | Switzerland | Not reported |
| Risk managements | | Kerse et al 2008 | New Zealand | Not reported |
| Mobility-Accessibility | Narsakka et al/2022 | de Boer et al. 2016 | Netherlands | Not reported |
| Technological Structure | | Braun et al., 2015 | Not reported | Not reported |
| | | Luyten et al., 2018 | Netherlands | Not reported |
| | | Lauzé et al., 2017 | Canada | Not reported |
| | | D'Cunha et al., 2020 | Australia | Not reported |
| Personnel Structure | | Stathi and Simey et al 2007 | UK | Not reported |
| | | Burke et al 2021 | UK | Not reported |
| | | Giné-Garriga et al. 2019 | Spain | Not reported |
| | | Hawkins et al., 2018 | UK | Not reported |
| | | MacDonald and Butler, 1974 | Incomplete Text | Incomplete Text |
| | | Holmes et al., 2017 | Not reported | Not reported |
| | | Yang et al., 2021 | Taiwan | Not reported |
| Physical structure | | Mahrs Träff et al., 2020 | Sweden | Not reported |
| | | Benjamin et al., 2009 | Canada | Not reported |
| | | Benjamin et al., 2011 | Canada | Private for profit/ Private non-profit |
| | | van Steenwinkel et al., 2017 | Belgium | Private non-profit |
| | | Baert et al., 2016 | Belgium | Public/Private for profit/ Private non-profit |
| | | Kalinowski et al., 2012 | Germany | Public/Private for profit/ Private non-profit |
| | | MacDonald, 2006 | Not reported | Not reported |
| | | Lemke e Moos, 1984 | Not reported | Not reported |

| | | | | |
|---|---|---|---|---|
| | | Hawkins et al., 2018 | UK | Not reported |
| | | Lu, 2010 | USA | Private for profit |
| | | Nordin et al., 2017 | Sweden | Not reported |
| | | Øye et al., 2017 | Norway | Not reported |
| | | Schwarz et al., 2004 | USA | Not reported |
| Pharmaceutical care | Morin et al/ 2016 | Bronskill et al 2012 | Canada | Private for profit |
| Mobility-Accessibility | Meulenbroeks et al/ 2022 | Kinley et al 2014 | UK | Not reported |
| Personnel Structure | | Bern-Klug et al 2013 | USA | Public/ Private for profit |
| | | Buddingh et 2013 | Not reported | Not reported |
| | | Giesbrecht et al 2012 | Canada | Public/Private for profit |
| | | Sterke et al 2021 | Netherlands | Not reported |
| Mobility-Accessibility | Maurer et al/2019 | Guerin et al., 2008 | Australia | Not reported |
| | | Lindelöf et al.,2017 | Sweden | Not reported |
| | | Behr et al., 2014 | Germany | Not reported |
| | | Taylor et al., 2013 | Australia | Private for profit |
| | | Bourret et al., 2002 | Canada | Not reported |
| | | Olsen et al., 2015 | Sweden | Not reported |
| Pharmaceutical care | Marasinghe et al/2015 | Donovan et al. 2010 | USA | Not reported |
| | | Field et al. 2009 | Canada | Not reported |
| | | Kennedy et al.2011 | Canada | Not reported |
| | | Tamblyn et al 2012 | Canada | Not reported |
| | | Judge et al 2006 | Not reported | Not reported |
| | | Handler et al 2008 | USA | Not reported |
| | | Gurwitz et al 2008 | Canada | Not reported |
| Personnel Structure | MäkiÿTurjaÿRostedt et al/2018 | Olsho et al., 2014 | USA | Not reported |
| Diet Food Services | | Pouyssegur et al., 2015 | France | Not reported |
| | | Brienza et al 2010 | USA | Not reported |

| | | | | |
|---|---|---|---|---|
| | | van Leen et al., 2014 | Netherlands | Not reported |
| | | Hampton & Collins, 2005 | Not reported | Not reported |
| Personnel Structure | Low et al. 2015 | Frenkel et al.2001 | Not reported | Not reported |
| | | van der Putten et al 2012 | Netherlands | Not reported |
| Risk managements | | Baldwin et al 210 | Northern Ireland | Public/ Private for profit |
| | | Ho et al 2012 | Not reported | Not reported |
| Personnel Structure | Liu et al/2015 | Coyne & Hoskins, 1997 | Not reported | Not reported |
| | | Van Ort & Phillips, 1995 | Incomplete Text | Incomplete Text |
| | | Simmons & Schnelle, 2004 | Not reported | Not reported |
| Physical structure | | Brush et al., 2002 | Incomplete Text | Incomplete Text |
| Risk managements | Lijas et al/2022 | Li et al 1996 | USA | Not reported |
| | | Rolland et al 2020 | France | Public/Private for profit/ Private non-profit |
| Personnel Structure | Li et al/2021 | Boakye-Dankwa et al 2017 | USA | Private for profit |
| | | Abrahamson et al 2020 | USA | Not reported |
| | | Liu et al 2014 | China | Private non-profit |
| Risk managements | Lee et al/2017 | Incomplete Text | Incomplete Text | Incomplete Text |
| Mobility-Accessibility | Kukkohov et al/2023 | Delbroek et al 2017 | Belgium | Not reported |
| | | Swinnen et al. 2021 | Belgium | Not reported |
| | | Mugueta-Aguinaga et al.2017 | Spain | Not reported |
| | | Cicek et al. 2020 | Turkey | Not reported |
| | | Fakhro et al. 2019 | Lebanon | Not reported |
| | | Yousef et al. 2021 | Iran | Not reported |
| | | Soares et al 2016 | Not reported | Not reported |
| | | Ramnath et al. 2021 | South Africa | Not reported |
| Technological Structure | | Mugueta-Aguinaga et al.2017 | Spain | Not reported |
| | | Soares et al 2016 | Not reported | Not reported |

| | | | | |
|---|---|---|---|---|
| | | Stanmore et al. 2019 | UK | Not reported |
| Risk managements | Konetzka et al/2021 | Telford et al 2021 | USA | Private for profit |
| Mobility-Accessibility | Kjelle et al/2017 | Laerum et al. 2005a | Incomplete Text | Incomplete Text |
| | | Forat et al. 2010 | Incomplete Text | Incomplete Text |
| | | Laerum et al. 2005b | Incomplete Text | Incomplete Text |
| Technological Structure | | Thingnes et al 2010 | Norway | Not reported |

Source: Prepared by the author

**Table S5.** Quality assessment of the systematic reviews selected in this overview (n= 61).

| Author/ Year | 1 | 2 | 3 | 4 | 5 | 6 | 7 | 8 | 9 | 10 | 11 | 12 | 13 | 14 | 15 | 16 | Quality of the Study |
|---|---|---|---|---|---|---|---|---|---|---|---|---|---|---|---|---|---|
| Bostick et al/2006 (11) | N | N | N | PY | N | N | N | N | N | Y | NA | NA | N | N | NA | N | Critially Low quality review |
| Sawka et al/2006 (12) | Y | Y | Y | PY | Y | Y | N | Y | N | Y | NA | NA | N | N | NA | N | Critially Low quality review |
| Dwyer et al/2011 (13) | Y | N | Y | N | Y | Y | Y | PY | Y | N | NA | NA | Y | Y | NA | N | Critially Low quality review |
| Neyens et al/2011 (14) | Y | N | Y | PY | Y | Y | N | Y | N | Y | NA | NA | Y | N | NA | N | Critially Low quality review |
| Sinclair et al/2011 (15) | N | N | N | N | N | N | N | N | N | N | NA | NA | N | N | NA | N | Critially Low quality review |
| Bradshaw et al/2012 (16) | N | N | Y | PY | N | N | N | Y | Y | Y | NA | NA | N | N | NA | N | Critially Low quality review |
| Tamura et al/2012 (17) | N | N | N | N | N | N | N | N | N | Y | NA | NA | N | N | NA | Y | Critially Low quality review |
| Valenzuela et al/2012 (18) | Y | N | Y | PY | N | N | N | Y | N | N | NA | NA | N | N | NA | N | Critially Low quality review |
| Abbott et al/2013 (19) | Y | Y | Y | PY | Y | Y | N | Y | Y | Y | Y | Y | Y | Y | Y | Y | Low quality review |
| Donald et al/2013 (20) | N | PY | Y | PY | Y | Y | Y | Y | N | Y | NA | NA | N | Y | NA | Y | Critially Low quality review |
| Edirippulige et al/2013 (21) | N | N | Y | PY | Y | Y | N | N | N | N | NA | NA | N | Y | NA | N | Critially Low quality review |
| Hughes et al/2013 (22) | Y | Y | Y | PY | Y | Y | Y | Y | Y | N | NA | NA | Y | Y | NA | N | Critially Low quality review |
| Reyes-Alcázar et al/2013 (23) | Y | Y | Y | PY | Y | Y | N | Y | N | Y | NA | NA | N | N | NA | Y | Critially Low quality review |
| Silva et al/2013 (24) | Y | Y | N | PY | N | Y | Y | Y | N | Y | Y | N | N | Y | Y | Y | Critially Low quality review |

| | | | | | | | | | | | | | | | | |
|---|---|---|---|---|---|---|---|---|---|---|---|---|---|---|---|---|
| Van Malderen et al/2013 (25) | N | N | N | PY | Y | Y | N | N | N | N | NA | NA | N | N | NA | N | Critially Low quality review |
| Chan et al/2014 (26) | Y | N | N | PY | Y | N | N | Y | PY | N | Y | Y | N | Y | Y | Y | Critially Low quality review |
| Flanagan et al/2014 (27) | Y | N | Y | N | Y | Y | N | Y | Y | Y | NA | NA | Y | N | NA | Y | Critially Low quality review |
| Bunn et al/2015 (28) | N | Y | Y | PY | Y | Y | N | Y | Y | Y | NA | NA | Y | Y | NA | Y | Low quality review |
| Liu et al/2015 (29) | Y | N | Y | PY | N | N | N | Y | N | N | NA | NA | N | N | NA | N | Critially Low quality review |
| Low et al/2015 (30) | Y | Y | Y | PY | Y | Y | N | Y | Y | Y | NA | NA | Y | Y | NA | Y | Low quality review |
| Marasinghe et al/2015 (31) | Y | N | Y | PY | Y | Y | N | Y | N | Y | NA | NA | N | N | NA | Y | Critially Low quality review |
| Pagan et al/2015 (32) | Y | N | Y | PY | Y | Y | N | PY | N | N | NA | NA | N | Y | NA | N | Critially Low quality review |
| Vlaeyen et al/2015 (33) | Y | Y | Y | PY | Y | Y | N | Y | Y | Y | Y | Y | N | Y | N | Y | Critially Low quality review |
| Alldred et al/2016 (34) | Y | PY | Y | PY | Y | Y | Y | Y | Y | Y | NA | NA | Y | Y | NA | Y | Moderate quality review |
| Diehl et al/2016 (35) | N | PY | Y | PY | Y | Y | Y | Y | Y | Y | NA | NA | Y | Y | NA | Y | Moderate quality review |
| Morin et al/ 2016 (36) | Y | Y | Y | PY | Y | Y | N | Y | Y | Y | Y | Y | Y | Y | Y | Y | Low quality review |
| Thiruchelvam et al/2016 (37) | Y | Y | Y | PY | Y | Y | N | Y | Y | N | NA | NA | Y | Y | NA | Y | Low quality review |
| Ferrah et al/2017 (38) | N | N | Y | N | N | N | N | Y | Y | N | NA | NA | Y | Y | NA | Y | Critially Low quality review |
| Kjelle et al/2017 (39) | Y | Y | Y | PY | Y | Y | N | Y | N | Y | NA | NA | N | N | NA | Y | Critially Low quality review |
| Lee et al/2017 (40) | Y | Y | Y | PY | Y | Y | N | Y | Y | Y | NA | NA | Y | Y | NA | N | Low quality review |

| | | | | | | | | | | | | | | | | |
|---|---|---|---|---|---|---|---|---|---|---|---|---|---|---|---|---|
| Barker et al/2018 (41) | Y | PY | N | PY | N | N | N | PY | Y | Y | NA | NA | Y | Y | NA | Y | Low quality review |
| Cao et al/2018 (42) | N | N | Y | PY | N | N | Y | PY | Y | Y | Y | Y | Y | Y | Y | Y | Critially Low quality review |
| Clarkson et al/2018 (43) | N | N | Y | PY | N | N | N | Y | Y | N | NA | NA | N | N | NA | N | Critially Low quality review |
| Ghavarskhar et al/2018 (44) | N | N | Y | PY | Y | N | N | N | N | Y | NA | NA | N | N | NA | Y | Critially Low quality review |
| MäkiÿTurjaÿ Rostedt et al/2018 (45) | Y | Y | Y | PY | Y | Y | N | Y | Y | Y | NA | NA | Y | Y | NA | Y | Low quality review |
| Panza et al/2018 (46) | Y | N | Y | PY | N | N | N | PY | N | N | NA | NA | N | Y | NA | N | Critially Low quality review |
| Brett et al/2019 (47) | Y | PY | Y | PY | Y | Y | N | Y | Y | Y | NA | NA | Y | N | NA | Y | Low quality review |
| Chen et al/ 2019 (48) | N | PY | Y | PY | Y | Y | Y | PY | Y | Y | NA | NA | N | N | NA | Y | Critially Low quality review |
| Donaldson et al/2019 (49) | Y | Y | Y | PY | Y | Y | N | Y | Y | Y | Y | Y | Y | Y | Y | Y | Low quality review |
| Huey Lee et al/2019 (50) | N | N | Y | PY | Y | N | N | PY | Y | N | Y | Y | Y | Y | Y | Y | Critially Low quality review |
| Hye Lee et al/2019 (51) | Y | PY | Y | PY | Y | Y | N | Y | Y | Y | NA | NA | Y | Y | NA | Y | Low quality review |
| Maurer et al/2019 (52) | Y | N | Y | PY | Y | Y | N | Y | N | N | NA | NA | N | N | NA | Y | Critially Low quality review |
| Nguyen et al/2019 (53) | Y | Y | Y | PY | Y | Y | N | Y | Y | Y | NA | NA | Y | Y | NA | Y | Low quality review |
| Schoberer et al/2019 (54) | Y | Y | Y | PY | Y | Y | N | Y | Y | Y | NA | NA | Y | Y | NA | Y | Low quality review |
| Senderovich et al/2019 (55) | Y | N | Y | N | N | N | N | N | N | Y | NA | NA | N | N | NA | Y | Critially Low quality review |
| van den Berg et al/2020 (56) | Y | N | Y | PY | Y | Y | N | N | N | Y | NA | NA | N | N | NA | Y | Critially Low quality review |
| Wu et al/2020 (57) | Y | Y | Y | PY | Y | Y | N | Y | Y | Y | NA | NA | Y | Y | NA | Y | High quality review |

| | | | | | | | | | | | | | | | | | |
|---|---|---|---|---|---|---|---|---|---|---|---|---|---|---|---|---|---|
| Ali et al/2021 (58) | Y | Y | Y | PY | Y | Y | N | Y | Y | Y | NA | NA | N | N | NA | Y | Low quality review |
| Frazer et al/2021 (59) | Y | Y | Y | N | Y | Y | N | PY | PY | Y | NA | NA | Y | Y | NA | Y | Critially Low quality review |
| Gonçalves et al/2021 (60) | Y | N | Y | PY | Y | Y | N | N | Y | Y | NA | NA | Y | N | NA | Y | Critially Low quality review |
| Huynh et al/2021 (61) | Y | N | N | PY | N | N | N | N | N | Y | NA | NA | N | N | NA | Y | Critially Low quality review |
| Konetzka et al/2021 (62) | Y | Y | Y | PY | Y | Y | N | Y | N | Y | NA | NA | N | N | NA | Y | Critially Low quality review |
| Li et al/2021 (63) | Y | N | Y | PY | Y | Y | N | Y | N | Y | NA | NA | Y | N | NA | N | Critially Low quality review |
| Wöhl et al/2021 (64) | Y | Y | Y | PY | Y | Y | N | Y | Y | Y | Y | Y | Y | Y | Y | Y | Low quality review |
| Chu et al/2022 (65) | Y | PY | Y | PY | Y | Y | N | Y | Y | Y | NA | NA | Y | N | NA | Y | Low quality review |
| Feehan et al/2022 (66) | Y | PY | Y | PY | N | N | N | Y | Y | Y | NA | NA | N | Y | NA | Y | Critially Low quality review |
| Lijas et al/2022 (67) | Y | N | Y | PY | Y | Y | N | Y | N | Y | NA | NA | Y | N | NA | N | Critially Low quality review |
| Meulenbroeks et al/2022 (68) | Y | Y | Y | PY | Y | Y | N | Y | Y | Y | NA | NA | N | Y | NA | Y | Critially Low quality review |
| Narsakka et al/2022 (69) | Y | Y | Y | PY | Y | Y | N | Y | Y | Y | NA | NA | Y | Y | NA | Y | Low quality review |
| Albasha et al/2023 (70) | Y | Y | Y | PY | Y | Y | Y | Y | Y | Y | NA | NA | Y | Y | NA | Y | Moderate quality review |
| Kukkohov et al/2023 (71) | Y | Y | Y | PY | Y | Y | N | Y | Y | Y | Y | Y | Y | N | Y | Y | Low quality review |

Y: Yes; N: No; NA: Not applicable
Source: Prepared by the authors

**Table S6.** Description of the factors related to the structure and functioning adopted globally for older adults highlighted in the outcomes mobility/accessibility, technological structure, personal structure, physical structure, pharmaceutical assistance, diet/food services and risk management.

| Author/Year | Mobility/accessibility |
|---|---|
| Valenzuela et al/2012 (22) | Functional exercise protocols improve measures of mobility, balance, flexibility and functional capacity. 1. Balance exercises and training showed significant improvements in muscle strength after a 10-week intervention. 2. Significant increases in muscle strength after six weeks of training the knee extensors at three different angles (90, 135, and 180) on a leg extension machine. 3. Muscle strength improved significantly (chest pressure and leg extension measurements) after eight weeks of low-intensity resistance training with dumbbells and shin weights. |
| Kjelle et al/2017 (43) | Mobile radiography services improved mobility and accessibility as 50-88% of residents would need ambulance transport and the remainder would need a wheelchair taxi, regular taxi or private car. |
| Cao et al/2018 (46) | Physical exercise improved the mobility of elderly people living in LTCF. |
| Maurer et al/2019 (56) | Exercising increases the autonomy of older people and being physically active helps them stay in shape, promotes social interaction and prevents isolation. Mobilization can promote physical function if the nurse is aware of the residents' resources and competencies. Two types of support allow them to participate in physical activities: 1) extrinsic motivators - family members or friends support participation in exercise programs; 2) activating aids can be a resource for being physically active: like a transfer board or walker. |
| Wöhl et al/2021 (68) | There was a small, statistically significant effect of physical activity compared to usual care or social activities in nursing homes on the viability of ATLs. |
| Chu et al/2022 (69) | In mobility and endurance results, 88% reported statistically significant improvements in the exergame group. Gait showed a 100% statistically significant improvement among the exergame intervention group. |
| Narsakka et al/2022 (73) | Residents in institutions with green areas, with more access to outdoor spaces and who perform more physical activities outdoors are significantly more physically active than residents in traditional nursing homes. |
| Kukkohov et al/2023 (75) | Significant positive changes in Timed Up and Go (TUG) test or Short Physical Performance Battery (SPPB) scores in the exergame group for mobility and accessibility. |

| Technological Structure | |
|---|---|
| Edirippulige et al/2013 (25) | Real-time telemedicine using videoconferencing was effective in reducing the number of clinic visits and achieved high resident satisfaction. Videoconferencing was considered a valuable alternative, especially for wound care and psychiatric diagnoses. |
| Bunn et al/2015 (32) | The Resident Assessment Instrument (RAI) is a comprehensive, standardized tool designed to assess residents living in long-term care and provides individualized care to promote functioning and prevent preventable illnesses. A reduction in the prevalence of dehydration was observed after the mandatory implementation of RAI in the United States between 1990 and 1991. |
| Marasinghe et al/2015 (35) | Computerized clinical decision support systems (CCDSS) improved the quality of prescribing decisions for physicians prescribing medications for LTCF residents with renal failure. The CCDSS with Computerized Physician Order Entry (CPOE) is effective in presenting alerts and represents a tool to improve medication safety. CCDSS with patient-specific risk estimates provides an effective method for reducing the risk of injury in vulnerable older adults. |
| Kjelle et al/2017 (43) | Mobile radiography services allowed an increase in the number of exams when it was implemented in nursing homes. |
| Ghavarskhar et al/2018 (48) | The provision of telecare and telehealth services through multiple systems helps with the basic features of assisted living services such as vital signs monitoring sensors to measure physiological parameters and teleconsultation services with caregivers using audio and video technologies. |
| Panza et al/2018 (50) | The use of mobile devices in nursing home residents may be able to perform an assessment of the elderly person's health status. Mobile devices: 1) self-Comprehensive geriatric assessment (CGA) with a modified MDS 3.0 converted to a format for use with a 6-inch mobile pad; 2) a 3.7-inch mobile smartphone. |

| | |
|---|---|
| Narsakka et al/2022 (73) | Technological solutions proved to be viable for monitoring psychogeriatric residents: 1) Interactive art induced physical responses in residents, especially when recognizable, such as projections of a football field or windmills, or seeing a child waving. 2) The exergame facilitated physical activity and was feasible to be used independently by residents after practice with a professional. 3) Virtual group cycling- pre-recorded video of an outdoor cycling trip where residents used pedal boards simultaneously facilitated residents' physical activity and was experienced as engaging. |
| Kukkohov et al/2023 (75) | The digital exergames FRED, SIRTET and MIRA system used on the Nintendo Wii and Microsoft Xbox-360 Kinect consoles were designed to improve balance, strength and flexibility. |
| **Personnel structure** | |
| Bostick et al/2006 (15) | Greater numbers of licensed Registered Nurse (RN) and Licensed Practical Nurse (LPN) employees are associated with higher quality. Increased RN time is also associated with better patient outcomes. An LPN number is required to evaluate situations and supervise unlicensed employees. |
| Dwyer et al/2011 (17) | Geriatric nurses are strongly motivated to work in the aged care sector and are passionate about producing a high level of quality care for older people. |
| Neyens et al/2011 (18) | Medication reviewed by a pharmacist reduces the number of falls per patient. |
| Bradshaw et al/ 2012 (20) | In cases where employees provided emotional or psychosocial care, understanding the residents' life history allowed them to be seen as people, promoting their self-esteem. Residents feel safe and confident with the continuity of the team. |
| Donald et al/2013 (24) | Advanced practice nurses improve or reduce cases of pressure ulcers. |
| Flanagan et al/2013 (31) | The majority of residents are physically dependent, requiring much more assistance from a professional, with the nursing assistant dedicating more time to helping the elderly person go to the bathroom. Standard nursing care improved hydration and decreased urinary incontinence in residents. |
| Liu et al/2015 (33) | Individual nursing assistance during feeding (e.g., verbal instructions and cues, positive reinforcement, praise, and appropriate encouragement) has been shown to be effective in improving feeding performance. Verbal assistance |

| | |
|---|---|
| | provided by nursing staff encouraged and engaged residents to continue with feeding tasks and thus possibly promoted self-feeding performance. |
| Low et al/2015 (34) | The oral health team improved residents' dental plaque among other oral health conditions. |
| Alldred et al/2016 (38) | Monthly multidisciplinary team meetings between the doctor, pharmacist and nurse were effective in discussing and improving the resident's medication use. |
| Barker et al/2018 (44) | Expert physician-based interventions are strongly associated with better prescribing outcomes. Including specialist professionals (doctors or nurses) in the primary care team improves key health outcomes for LTCF residents. Positive association between the inclusion of a specialist doctor and a reduction in unplanned hospital transfers. |
| Clarkson et al/2018 (47) | Pharmacy staff have helped reduce the number of inappropriate medications prescribed with associated cost reductions, including a reduction in resident falls. Advanced practice nurses contributed to the reduction of urinary/fecal incontinence, pressure ulcers, and aggressive behavior among residents. |
| Ghavarskhar et al/2018 (48) | Nursing care assists with activities of daily living (monitoring weight and blood glucose, vital signs, treating wounds, treating pain, managing medications and applying intramuscular and intravenous injections). Service providers in nursing homes included nurses, nursing assistants, general practitioners, rehabilitation specialists, social workers, psychiatrists, psychologists, dentists, and chiropodists. |
| MäkiÿTurjaÿRostedt et al/2018 (49) | When using weekly reports, the nursing team significantly reduced the incidence of Pressure Ulcers. |
| Panza et al/2018 (50) | Nurse specialists in gerontology from the Residential Care Integration Program for the Elderly (RACIP) reduced the hospitalization rate in LTCF residents. |
| Brett et al/2019 (51) | The use of physiotherapy services can be attributed to the strong rehabilitation approach adopted in nursing homes as an allied contribution to health. |

| | |
|---|---|
| Huey Lee et al/2019 (54) | Most services provided by pharmacists aimed to address medication safety among residents, but there was considerable diversity in how these services were delivered, including clinical medication review, provider education, multidisciplinary team meetings, and multifaceted interventions. |
| Nguyen et al/2019 (57) | The nursing team was in compliance with an antimicrobial administration tool that did not present any unexpected adverse effects guiding care for the elderly person. |
| Wu et al/2020 (61) | Implementation of an antimicrobial stewardship intervention led by a clinical nurse consultant led to a statistically significant increase in reduction in antibiotic use. After hiring a nurse there were statistically significant improvements in managing asymptomatic urinary tract infections. Positive effects of the intervention with increased fluid intake and frequency of bathroom use. |
| Ali et al/2021 (62) | Pharmacists have significantly helped reduce adverse drug reactions. |
| Gonçalves et al/2021 (64) | The pharmacy professional used a clinical informatics tool developed by a pharmaceutical association (American Society of Consultant Pharmacists) to reduce negative adverse drug reactions. Pharmacist developed an algorithm to signal residents about increased risk for medication-related problems. |
| Li et al/2021 (67) | Nursing care in long-term care facilities was considered comprehensive and personal, including medical, psychosocial, and spiritual needs, as well as maintenance of the living environment. Nursing Assistants (NA) were described as versatile, playing a central role in providing long-term care with support for pain management. |
| Meulenbroeks et al/2022 (72) | Physiotherapy was associated with better scores in activities of daily living at six months of follow-up. A one-hour increase in physical and occupational therapy staff improved activities of daily living. Physiotherapists worked to prevent falls, provide advice on mobility transfers and treat pressure ulcers. Increasing one occupational therapist per 100 residents reduced the likelihood of wheelchair seating issues by 90%. Social workers participated in quarterly care plan meetings and provided emotional support to families. Nutritionist was associated with high food satisfaction among residents. |

| | |
|---|---|
| Narsakka et al/2022 (73) | The opinions of health professionals and the residents' trust in them were important for the encouragement and social support that facilitated the execution of daily activities. |
| Albasha et al/2023 (74) | The self-efficacy of the nursing team had a positive impact. |
| **Physical structure** | |
| Van Malderen et al/2013 (29) | The Casa Verde concept with more vegetation, trees and flowers with outdoor spaces and providing a more family-friendly dining area led to an improvement in the quality of life of residents. |
| Liu et al/2015 (33) | The bright environment, contrast of table layout, and family-style meal delivery were effective in improving eating performance. Environmental and routine modifications can improve resident comfort during meals and can be easily implemented by LTCF professionals. |
| Ghavarskhar et al/2018 (48) | Emergency alarm, flood detector and smoke sensors are required in case of fire in nursing homes. |
| van den Berg et al/2020 (60) | Vegetation and garden features should allow residents to garden, touch, see, smell and discuss vegetation with others. Trees were seen as important for providing shade and a sense of peace through contact with nature. Interaction with fauna was a positive aspect in outdoor spaces, such as watching and listening to birds and seeing butterflies in the garden. Water features like ponds were also appreciated for their aesthetic appeal and calming effect. These locations cannot be far from the main residence.<br>Paths and surfaces that were steep, inclined, slippery, very narrow, had uneven surfaces, or were difficult to access from the main dwelling represented a danger to residents (especially those using wheelchairs or walkers) and restricted the use of outdoor areas .<br>Seating was used most frequently in outdoor spaces. Seats must be sufficient, easily accessible and comfortable.<br>Doors should not be too heavy or locked so as not to be a barrier to independent access if outdoors. Automatic doors are preferable to manual doors, but the automatic locking mechanism is prevented.<br>Stops or thresholds should not be high, as they impede the movement of a wheelchair or walker. |

| | |
|---|---|
| | Residents' rooms must be on the ground floor, so as not to cause barriers to outside access.<br>Shade and shelter in outdoor areas should be provided such as gazebos, terraces, pergolas or roofs, protecting residents from sun, wind and rain.<br>Outdoor spaces should be lit, especially at night. |
| Feehan et al/2022 (71) | Spending time outdoors, exposed to sunlight, was important for synthesizing vitamin D. |
| Narsakkaet al/ 2022 (73) | Locked, heavy and difficult doors limited residents' freedom of movement.<br>Sufficiently wide doors and automatic doors/elevators were considered important for residents.<br>Door sills, paths and steep ramps prevented residents from moving around.<br>The characteristics of flooring materials such as gloss and color differences were limiting for residents due to sensory distractions.<br>Well-lit areas made walking easier for residents.<br>Seats for resting in outdoor areas must have shade, handrails and adequate signage to help with movement.<br>Staff areas should be close to resident bedrooms with visual access to open-plan hallways and glazed courtyards.<br>Monitoring devices such as wireless alarm systems have brought ease and speed in resolving cases. |
| **Pharmaceutical care** | |
| Tamura et al/2012 (21) | The chances of polypharmacy decreased with the increase in the number of beds, observing the following classification: small facilities (3–99 beds), medium facilities (100–199 beds) and large facilities (200 beds or more). |
| Reyes-Alcázar et al/2013 (27) | Individualized medication records. Periodic medication reviews by authorized staff. Self-administration of medications with specific permissions. Dispensing in original containers. Storage system adjusted to health surveillance regulations. Maturity control system. Establishment of standards for administration of alternative medicines and administration of emergency medicines under medical advice. Electronic devices to improve medication recording and management. |

| | |
|---|---|
| Ferrah et al/2016 (42) | The majority of medication errors occurred in the administration and order communication phases (20–53%) and in prescribing and monitoring (59–100%). LTCFs with a capacity greater than 150 beds had twice the medication error rate (number of medication errors per bed over 1 year) as smaller facilities. |
| Morin et al/ 2016 (40) | Higher rates of polypharmacy have been correlated with the prevalence of potentially inappropriate medication use. |
| Thiruchelvam et al/2016 (41) | Pharmaceutical assistance helped in suspending drug therapy (30%), reducing dosage (28%) and changing generics and tests to monitor drug therapy (22%). The most common recommendation was to discontinue medications (1.7 medications discontinued for each resident reviewed). Medication review is an essential component of resident-centered care. |
| Chen et al/ 2019 (52) | Medication reviews helped with optimization by decreasing inappropriate prescribing and use of prescription anticholinergics or sedatives. Comprehensive medication reviews have been successful in identifying medication-related problems per resident in up to 84% of recommendations. |
| Huey Lee et al/2019 (54) | Pharmaceutical services in nursing homes have improved prescription quality. The medication review had a positive impact on quality of life, reducing mortality and hospitalization rates. |
| Ali et al/2021 (62) | Medication reviews reduced the risks of inadequate treatment in cases of cystitis, use of anticholinergic medications, falls, cognitive impairment and delirium. |
| **Diet/Food Services** | |
| Neyens et al/2011 (18) | Calcium supplementation plus vitamin D were effective in reducing falls in residents. |
| Sinclair et al/2011 (19) | All elderly people entering the nursing home must be screened for malnutrition. |
| Abbott et al/2013 (23) | Nursing home residents consumed significantly more energy (17 kcal (7%)/meal) when sauce was added to their meals compared to meals served without sauce. The sauces used in the study were meat sauce, mustard sauce, white sauce and vegetable-based sauce. Providing real food snacks resulted in 20–25% higher caloric intake. |

| | |
|---|---|
| Flanagan et al/2013 (31) | Routine health care using hydration carts and written procedures improved hydration and decreased incontinence in residents. Regular guidance and fluid assistance incorporated into routine care can have a positive effect on maintaining resident continence and hydration, with potential benefits for overall health, well-being and quality of life. |
| Bunn et al/2015 (32) | Increased fluid intake was associated with greater availability and choice of beverages. Offering 5 meals a day to dysphagic elderly people increased their daily fluid intake. |
| Thiruchelvam et al/2016 (41) | High-dose vitamin D3 supplementation resulted in significantly fewer acute respiratory infections per person-year and a higher incidence of falls in residents than the standard dose. |
| MäkiÿTurjaÿRostedt et al/2018 (49) | The nutritional diet significantly reduced the prevalence of pressure ulcers. Subgroup analysis confirmed the positive impact of isolated Protibis* biscuit supplementation in reducing pressure ulcers. |
| Panza et al/2018 (50) | The Rapid Geriatric Assessment was successfully used in more than 1,500 elderly people, providing assistance with nutritional diet. |
| Donaldson et al/2019 (53) | High protein intake significantly increased the average Body Mass Index (BMI) in residents. |
| Feehan et al/2022 (70) | Supplementation was effective in improving vitamin D performance contributing to the prevention of vitamin D insufficiency, having a vitamin D status <50 nmol/L. Vitamin D3 supplementation should be encouraged over vitamin D2 as it potentially leads to sufficiency more quickly. However, vitamin D supplementation in very high doses results in some adverse effects in older people, including increased risk of falls and fractures; therefore, standard clinical practice is to administer more modest doses daily or weekly. |
| **Risk management** | |
| Sawka et al/2006 (16) | Hip protectors decreased the risk of hip fracture in residents at least twice as much when compared to no intervention. |
| Neyens et al/2011 (18) | The fall prevention program showed that the group with greater cognition benefits more in terms of reducing falls and the group with lower cognition benefits more in terms of preventing fractures. Therefore, team education was very important for preventing falls. |

| | |
|---|---|
| Hughes et al/2013 (26) | Infection control audits relating hand hygiene and equipment decontamination were effective in preventing MRSA transmission. |
| Silva et al/2013 (28) | Exercise was the main intervention for preventing and reducing falls in LTCFs. The effect of exercise improves strength, endurance, muscular flexibility and postural balance, reducing physical disability and functional limitations in the elderly and helping them maintain compensatory mechanisms against an imminent fall. Combined exercise increases muscle strength, balance and mobility, making it an effective intervention to reduce falls regardless of the clinical scenario. Exercise programs were effective in preventing falls if carried out for 1 to 3 months or more than 6 months with a frequency of 2 to 3 times a week, while programs that lasted between 3 and 6 months showed no significant effectiveness. |
| Chan et al/2014 (30) | Influenza vaccination significantly reduced pneumonia, death from pneumonia or flu among residents. |
| Low et al/2015 (34) | Staff behavior improved infection control and hand hygiene in residents by reducing hospitalization related to methicillin-resistant Staphylococcus aureus (MRSA) or respiratory outbreaks. |
| Pagan et al/2015 (36) | Implementation of programs incorporating an algorithm to guide pressure injury interventions has demonstrated a significant reduction in the rate of pressure injury with relief support to reduce the risk of wound worsening. |
| Vlaeyen et al/2015 (37) | Exercise, medication, physical environment, hip protectors, footwear, goal setting, reminders and feedback showed a significant beneficial effect of the intervention in relation to the number of falls. |
| Diehl et al/2016 (39) | The implementation of the patient safety program “SAFE OR SORRY? ” significantly reduced adverse events and aided in preventative care for residents at risk of pressure ulcers, urinary tract infections or falls. |
| Lee et al/2017 (44) | Exercises (combined) involving gait, balance and functional training with mechanical and strength devices reduced the rate of falls. Balance with single-leg functional training and Tai Chi did not differ significantly, but tended to reduce the rate of falls. |
| Kjelle et al/2017 (43) | Mobile radiography services in the nursing home facilitated pneumonia treatment with a 6% reduction in hospitalization and an 11.5% reduction in emergency room visits for residents. |

| | |
|---|---|
| MäkiÿTurjaÿRostedt et al/2018 (49) | The air cushion, viscous fluid and foam or gel and foam cushion significantly decreased the incidence of pressure ulcers (PU) near the ischial tuberosities. Replacing the standard viscoelastic mattress with a static air mattress in signs of UP. If signs of PU still persisted, the resident's mattress was replaced with a low air loss system. Pressure injuries have also been reduced by switching from standard mattresses to memory foam mattresses and memory foam pillows. |
| Hye Lee et al/2019 (55) | The World Health Organization's (WHO) multimodal strategy with four or more elements was associated with a decrease in respiratory infections or Multidrug Resistant Organisms (MDRO) infections, which resulted in better adherence to hand hygiene care. |
| Schoberer et al/2019 (58) | Exercises with a balance component significantly reduced the number of falls. Exercises performed with technical and guided devices showed a significantly beneficial pooled effect. |
| Senderovich et al/2019 (59) | Before vaccination, the immunity of LTCF residents was significantly more compromised. After administration of the vaccine, the immunity conferred on LTCF residents was similar to that of elderly people living in the community. |
| Frazer et al/2021 (63) | Mass testing, including isolation of infected residents with reassignment of specific care personnel, hand hygiene, contact precautions for residents and LTCF staff were the main measures used to reduce the transmission of COVID-19. Restricting visitor access to facilities was implemented to reduce the likelihood of COVID-19 transmission in LTCFs by assessing body temperature and screening for symptoms of staff and visitors. The likelihood of having a case of COVID-19 increased in medium and large facilities compared to small facilities. Facilities with high capacity had more infections and deaths than those with low capacity. Simulations carried out suggested that almost 20% of infections and deaths could have been avoided by converting all four-bed rooms into two-bed rooms. |

| | |
|---|---|
| Huynh et al/2021 (65) | Implementing fall prevention policies with bed rails can serve as a tool to provide comfort to residents. Bed rails can help reposition residents by preventing them from accidentally rolling out of bed. However, bed rails are not the most effective fall prevention tool, as residents can climb onto the rails putting them at risk of more serious injuries. Another strategy includes lowering beds to reduce the risk of falls or minimize potential harm. |
| Konetzka et al/2021 (66) | Facilities with fewer COVID-19 cases had better practices related to Personal Protective Equipment (PPE) and social distancing. |
| Lijas et al/2022 (71) | The COVID-19 pandemic showed that sectoring professionals within zones resulted in a significantly lower probability of having any confirmed cases of COVID-19, with the risks being 2.5 times greater for outbreaks of nosocomial respiratory or gastrointestinal illnesses if the care team worked across multiple units. |

*Protibis: wheat flour, fresh butter, milk protein (casein), sugar, vanilla flavor, baking powder and salt.

Source: Authors' elaboration
